# Aromatase inhibitors versus tamoxifen in premenopausal women with oestrogen receptor-positive early-stage breast cancer treated with ovarian suppression: a patient-level meta-analysis of 7030 women from four randomised trials

**DOI:** 10.1016/S1470-2045(21)00758-0

**Published:** 2022-03

**Authors:** Early Breast Cancer Trialists' Collaborative Group (EBCTCG), Rosie Bradley, Jeremy Braybrooke, Richard Gray, Robert K Hills, Zulian Liu, Hongchao Pan, Richard Peto, David Dodwell, Paul McGale, Carolyn Taylor, Prudence A Francis, Michael Gnant, Francesco Perrone, Meredith M Regan, Richard Berry, Clare Boddington, Mike Clarke, Christina Davies, Lucy Davies, Fran Duane, Vaughan Evans, Jo Gay, Lucy Gettins, Jon Godwin, Sam James, Hui Liu, Elizabeth MacKinnon, Gurdeep Mannu, Theresa McHugh, Philip Morris, Simon Read, Ewan Straiton, Raimund Jakesz, Christian Fesl, Olivia Pagani, Richard Gelber, Michelino De Laurentiis, Sabino De Placido, Ciro Gallo, Kathy Albain, Stewart Anderson, Rodrigo Arriagada, John Bartlett, Elizabeth Bergsten-Nordström, Judith Bliss, Etienne Brain, Lisa Carey, Robert Coleman, Jack Cuzick, Nancy Davidson, Lucia Del Mastro, Angelo Di Leo, James Dignam, Mitch Dowsett, Bent Ejlertsen, Matthew Goetz, Pam Goodwin, Pat Halpin-Murphy, Dan Hayes, Catherine Hill, Reshma Jagsi, Wolfgang Janni, Sibylle Loibl, Eleftherios P Mamounas, Miguel Martín, Hirofumi Mukai, Valentina Nekljudova, Larry Norton, Yasuo Ohashi, Lori Pierce, Philip Poortmans, Kathleen I Pritchard, Vinod Raina, Daniel Rea, John Robertson, Emiel Rutgers, Tanja Spanic, Joseph Sparano, Guenther Steger, Gong Tang, Masakazu Toi, Andrew Tutt, Giuseppe Viale, Xiang Wang, Tim Whelan, Nicholas Wilcken, Norman Wolmark, David Cameron, Jonas Bergh, Sandra M Swain

## Abstract

**Background:**

For women with early-stage oestrogen receptor (ER)-positive breast cancer, adjuvant tamoxifen reduces 15-year breast cancer mortality by a third. Aromatase inhibitors are more effective than tamoxifen in postmenopausal women but are ineffective in premenopausal women when used without ovarian suppression. We aimed to investigate whether premenopausal women treated with ovarian suppression benefit from aromatase inhibitors.

**Methods:**

We did a meta-analysis of individual patient data from randomised trials comparing aromatase inhibitors (anastrozole, exemestane, or letrozole) versus tamoxifen for 3 or 5 years in premenopausal women with ER-positive breast cancer receiving ovarian suppression (goserelin or triptorelin) or ablation. We collected data on baseline characteristics, dates and sites of any breast cancer recurrence or second primary cancer, and dates and causes of death. Primary outcomes were breast cancer recurrence (distant, locoregional, or contralateral), breast cancer mortality, death without recurrence, and all-cause mortality. As distant recurrence invariably results in death from breast cancer several years after the occurrence, whereas locoregional recurrence and new contralateral breast cancer are not usually fatal, the distant recurrence analysis is shown separately. Standard intention-to-treat log-rank analyses estimated first-event rate ratios (RR) and their confidence intervals (CIs).

**Findings:**

We obtained data from all four identified trials (ABCSG XII, SOFT, TEXT, and HOBOE trials), which included 7030 women with ER-positive tumours enrolled between June 17, 1999, and Aug 4, 2015. Median follow-up was 8·0 years (IQR 6·1–9·3). The rate of breast cancer recurrence was lower for women allocated to an aromatase inhibitor than for women assigned to tamoxifen (RR 0·79, 95% CI 0·69–0·90, p=0·0005). The main benefit was seen in years 0–4 (RR 0·68, 99% CI 0·55–0·85; p<0·0001), the period when treatments differed, with a 3·2% (95% CI 1·8–4·5) absolute reduction in 5-year recurrence risk (6·9% *vs* 10·1%). There was no further benefit, or loss of benefit, in years 5–9 (RR 0·98, 99% CI 0·73–1·33, p=0·89) or beyond year 10. Distant recurrence was reduced with aromatase inhibitor (RR 0·83, 95% CI 0·71–0·97; p=0·018). No significant differences were observed between treatments for breast cancer mortality (RR 1·01, 95% CI 0·82–1·24; p=0·94), death without recurrence (1·30, 0·75–2·25; p=0·34), or all-cause mortality (1·04, 0·86–1·27; p=0·68). There were more bone fractures with aromatase inhibitor than with tamoxifen (227 [6·4%] of 3528 women allocated to an aromatase inhibitor *vs* 180 [5·1%] of 3502 women allocated to tamoxifen; RR 1·27 [95% CI 1·04–1·54]; p=0·017). Non-breast cancer deaths (30 [0·9%] *vs* 24 [0·7%]; 1·30 [0·75–2·25]; p=0·36) and endometrial cancer (seven [0·2%] *vs* 15 [0·3%]; 0·52 [0·22–1·23]; p=0·14) were rare.

**Interpretation:**

Using an aromatase inhibitor rather than tamoxifen in premenopausal women receiving ovarian suppression reduces the risk of breast cancer recurrence. Longer follow-up is needed to assess any impact on breast cancer mortality.

**Funding:**

Cancer Research UK, UK Medical Research Council.

## Introduction

For women with early-stage hormone receptor-positive breast cancer, adjuvant treatment with 5 years of the selective oestrogen receptor modulator tamoxifen reduces their risk of death from breast cancer at 15 years by about one third.^1^ Aromatase inhibitors are, for postmenopausal women, an even more effective endocrine treatment than tamoxifen, with further proportional reductions in recurrence rates of about 30%.^2^ Aromatase inhibitors, which block the conversion of androgens into oestrogens, are ineffective in premenopausal women, in the absence of ovarian suppression, because compensatory physiological responses induce ovarian oestrogen production. However, in premenopausal women treated with ovarian function suppression or ablation, another efficacious endocrine treatment,^3^ this physiological response is overcome and aromatase inhibitors might, therefore, also be more efficacious than tamoxifen at preventing breast cancer recurrence.^4^ To test this hypothesis, at least four randomised trials have compared aromatase inhibitors with tamoxifen in premenopausal women receiving ovarian suppression or ablation but with conflicting results.^5^, ^6^, ^7^, ^8^, ^9^ This collaborative meta-analysis of individual patient data from these trials aims to better define the benefits and risks of aromatase inhibitors compared with tamoxifen in women who have their ovarian function suppressed.

**Research in context**

**Evidence before this study**

A previous Early Breast Cancer Trialists' Collaborative Group (EBCTCG) meta-analysis of trials of aromatase inhibitors versus tamoxifen in postmenopausal women with early-stage oestrogen receptor (ER)-positive breast cancer has shown that aromatase inhibitors reduce recurrence rates by about 30% compared with tamoxifen over the 5-year treatment period. Aromatase inhibitors have not been shown to be efficacious in premenopausal women; however, premenopausal women treated with ovarian suppression might benefit from aromatase inhibitors. The EBCTCG's ongoing extensive searches of bibliographic databases, including MEDLINE, Embase, the Cochrane Library, and meeting abstracts, from database inception to July 31, 2020, identified four trials comparing aromatase inhibitors with tamoxifen in premenopausal women receiving ovarian suppression. Individual trials reported inconsistent results.

**Added value of this study**

This collaborative meta-analysis collated, checked, and analysed individual patient-level data from 7030 women in the four randomised trials. Aromatase inhibitors reduced the rate of breast cancer recurrence compared with tamoxifen. The absolute reduction in the 5-year risk of breast cancer recurrence was 3·2%, but no difference was apparent in breast cancer mortality. Few non-breast cancer deaths occurred.

**Implications of all the available evidence**

For premenopausal women with early-stage, ER-positive breast cancer treated with ovarian suppression, use of an aromatase inhibitor rather than tamoxifen in premenopausal women receiving ovarian suppression reduces the risk of breast cancer recurrence.

## Methods

### Study design and participants

Methods of identifying trials, data collection, checking, analysis, and presentation have been described in previous EBCTCG reports,^2^, ^10^, ^11^, ^12^ and conform to the Preferred Reporting Items for Systematic Review and Meta-Analyses (Individual Patient Data).^13^

Trials were eligible if they began before Jan 1, 2010, and randomly assigned premenopausal women with oestrogen receptor (ER)-positive operable breast cancer (defined as ER expression in at least 1% of tumour cells, by immunohistochemistry in SOFT,^7^, ^8^ TEXT,^7^, ^8^ and HOBOE;^9^ ABCSG XII^5^, ^6^ used Reiner score for staining of tumour-cell nuclei to define ER positivity [score ≥3; ie, ≥10% cells are positive]) to an aromatase inhibitor plus ovarian function supression versus tamoxifen plus ovarian function supression. Most trials included a small number of women with ER-negative, progesterone receptor (PR)-positive tumours but these individuals were excluded from this meta-analysis because the benefits of endocrine therapy are uncertain for such women.^1^ Between 2018 and 2021, we requested individual patient-level data from trial groups on randomisation date; allocated treatment; use of chemotherapy; age; body-mass index (BMI); tumour diameter; tumour grade; histology; involvement of locoregional lymph nodes; ER and PR status; human epidermal growth factor receptor 2 (HER2) status; and dates of any locoregional, contralateral, or distant breast cancer recurrence, other second primary cancer (including endometrial cancer), bone fracture, and death; and cause of death.

The primary outcomes were any recurrence of invasive breast cancer (distant, locoregional, or new primary in the contralateral breast), breast cancer mortality, death without recurrence, and all-cause mortality. As distant recurrence invariably results in death from breast cancer several years after the occurrence, whereas locoregional recurrence and new contralateral breast cancer are not usually fatal, the distant recurrence analysis is shown separately. Secondary outcomes were incidence and site of second primary cancers (including endometrial cancer), and bone fracture. Prespecified primary subgroup investigations for any recurrence, distant recurrence, and breast cancer mortality were follow-up period (years 0–1, 2–4, 5–9, and ≥10), site of recurrence, age, BMI, nodal status, PR status, tumour diameter, histological tumour grade, histology (ductal or lobular), HER2 status, use or not of neoadjuvant or adjuvant chemotherapy, and use or not of bisphosphonate.

### Statistical analysis

Statistical methods (stratified log-rank statistics, Kaplan-Meier graphs) are described in previous EBCTCG reports,^2^, ^10^, ^11^, ^12^ and in the statistical analysis plan (appendix). Briefly, time-to-event analyses were stratified by age, nodal status, and trial. Each analysis compared all women randomised, regardless of treatment compliance (intention-to-treat analyses). Log-rank statistics were used to compare the treatment effects (aromatase inhibitor versus tamoxifen) on all outcomes (primary and secondary), and, for each outcome, to estimate first-event rate ratios (RRs) and their 95% CIs. If a log-rank statistic (observed [o]–expected [e]) has variance v, then, defining z=(o–e)/sqrt(v) and b=(o–e)/v, where b has variance 1/v, the outcome RR (aromatase inhibitor *vs* tamoxifen) is estimated as exp(b) with SE=(RR–1)/z. 95% CIs and 99% CIs for RRs are derived from those for b (by normal approximations). Two-sided significance with p values of less than 0·05 were considered significant for analyses of the primary and secondary outcomes and, to compensate for multiple investigations, p values of less than 0·01 were considered significant for subgroup analyses. 95% CIs were estimated for meta-analyses, and 99% CIs were estimated for individual trials or subgroups. χ^2^ tests are used to assess tests for heterogeneity and test for trends in subgroup analyses.^11^ Breast cancer mortality RRs are estimated by subtracting log-rank statistics for mortality without recurrence from those of overall mortality, which avoids the need to determine which deaths after recurrence were from breast cancer.

If, as observed in the EBCTCG meta-analysis^2^ of aromatase inhibitors versus tamoxifen in postmenopausal women, almost all of the benefits of aromatase inhibitors over tamoxifen were seen in the period when the treatments differed, then subgroup analyses of any recurrence just in this period would also be undertaken as a post-hoc analysis to enhance statistical power to investigate any variability in treatment efficacy by patient or tumour characteristics. We also did post-hoc analyses comparing the nodal status subgroup finding for premenopausal women with those we have previously reported for aromatase inhibitor versus tamoxifen in postmenopausal women. Data from premenopausal and postmenopausal will also be combined to show the effects by nodal status overall. Forest plots and Kaplan-Meier graphs describe the separate trials and their combined results, and subgroup analyses explore whether proportional risk reductions depend on patient or tumour-related characteristics. In-house FORTRAN programs were used for statistical analyses.

### Role of the funding source

The funders of the study had no roles in the study design, data collection, data analysis, data interpretation, or writing of the report.

## Results

Individual patient data were provided for all four identified, relevant trials,^5^, ^6^, ^7^, ^8^, ^9^ including 7230 premenopausal women receiving ovarian suppression or ablation, who were enrolled between June 17, 1999, and Aug 4, 2015, and randomised between an aromatase inhibitor and tamoxifen (table). This report is restricted to 7030 (97·2%) women with ER-positive tumours. 4231 (60·2%) of 7030 women had node-negative cancer. Women in SOFT, TEXT, and HOBOE were randomly assigned to either 5 years of an aromatase inhibitor or 5 years of tamoxifen, whereas women in ABCSG XII were randomly assigned to 3 years of an aromatase inhibitor or 3 years of tamoxifen, with or without zoledronic acid. The aromatase inhibitor used was anastrozole in ABCSG XII, exemestane in SOFT and TEXT, and letrozole in HOBOE. HOBOE also included a letrozole plus zoledronic acid group, which was excluded from these analyses because was no control group receiving tamoxifen and zoledronic acid. Use of bisphosphonates for declining bone density was optional in SOFT and TEXT; however, the routine use of bisphosphonates was not permitted and only a minority of women reported bisphosphonate use during adjuvant therapy.^8^

**Table:** Baseline patient characteristics by trial

	**Years of recruitment**	**Number of patients**	**Key eligibility criteria of trial**	**Treatment comparison**	**Proportion of patients who received chemotherapy (%) and timing**	**Surgery type**	**Median age (IQR), years**	**Nodal status**	**Tumour size**	**Tumour grade**	ER status*	**HER2 status**	**Median follow-up (IQR), years**
ABCSG XII^5^, ^6^	1999–2006	1803	Aged 19–59 years; ER positive, PR positive, or both by Reiner score; <10 positive lymph nodes; exclusion: T1a tumours	Goserelin: (anastrozole *vs* tamoxifen) with or without zoledronic acid for 3 years	5·7% of patients received neoadjuvant chemotherapy before randomisation	80% mastectomy; 18% partial mastectomy; 2% unknown	45 (41–48)	67% N0; 27% N1–3; 4% N4–9; 2% unknown	75% T1; 20% T2; 1% T3–4; 4% unknown	14% well differentiated; 62% moderately differentiated; 20% poorly differentiated; 4% unknown	4% ER-negative; 93% ER-positive; 2% unknown	No available data	8·0 (7·8– 8·1)
TEXT^7^, ^8^	2003–11	2672	ER positive, PR positive, or both by IHC (≥10%)	Triptorelin: (exemestane *vs* tamoxifen) for 5 years	60% of patients received chemotherapy, which was optional; if administered, it was given concurrently with triptorelin; AI or tamoxifen started after completion of chemotherapy	59% partial mastectomy; 41% mastectomy	43 (40–46)	52% N0; 34% N1–3; 10% N4–9; 4% N10 or higher	59% T1; 36% T2; 4% T3–4; 1% unknown	17% well differentiated; 55% moderately differentiated; 27% poorly differentiated; 1% unknown	1% ER-negative; 99% ER-positive	85% HER2-negative; 14% HER2-positive; 1% unknown	9·1 (6·3– 10·2)
SOFT^7^, ^8^	2003–11	2045	ER positive, PR positive, or both by IHC (≥10%)	Triptorelin: (exemestane *vs* tamoxifen) for 5 years	53% of patients received adjuvant or neoadjuvant chemotherapy, which was allowed before randomisation, as long as patient remained premenopausal after completion of chemotherapy	58% partial mastectomy; 42% mastectomy	43 (38–47)	65% N0; 24% N1–3; 8% N4–9; 2% N10 or higher; 1% unknown	65% T1; 28% T2; 4% T3–4; 3% unknown	25% well differentiated; 53% moderately differentiated; 20% poorly differentiated; 2% unknown	2% ER-negative; 98% ER-positive	86% HER2-negative; 13% HER2-positive; 1% unknown	7·9 (6·2– 9·2)
HOBOE^9^	2004–15	710	Aged 18 years or older; ER positive, PR positive, or both by IHC (≥1%)	Triptorelin: (letrozole *vs* tamoxifen) for 5 years†	63% of patients received neoadjuvant chemotherapy, adjuvant chemotherapy, or both; chemotherapy allowed before randomisation	74% partial mastectomy; 25% modified radical mastectomy	44 (41–48)	55% N0; 31% N1–3; 10% N4–9; 4% N10 or higher	68% T1; 27% T2; 3% T3–4; 2% unknown	10% well differentiated; 52% moderately differentiated; 34% poorly differentiated; 4% unknown	1% ER-negative; 99% ER-positive	85% HER2-negative; 14% HER2-positive; 1% unknown	5·3 (3·7– 7·2)

* ER positivity in this meta-analysis is defined as expression in at least 1% of tumour cells by immunohistochemistry in SOFT, TEXT (by central assessment if available and local assessment otherwise), and HOBOE; ABCSG 12 used the Reiner score for staining of tumour-cell nuclei to define expression levels (on a scale of 10–100%, with 10–50% indicating low expression, 51–80% indicating medium expression, and 81–100% indicating high expression).

† A separate group of patients given letrozole plus zoledronic acid were excluded from the analysis. AI=aromatase inhibitor. ER=oestrogen receptor. IHC=immunohistochemistry. PR=progesterone receptor.

Ovarian function supression was achieved with triptorelin in SOFT, TEXT, and HOBOE, and goserelin in ABCSG XII, all at licensed doses for the duration of endocrine therapy. In SOFT (and TEXT after at least 6 months), ovarian suppression could, alternatively, be achieved by bilateral oophorectomy or ovarian irradiation. In HOBOE, neoadjuvant or adjuvant chemotherapy was allowed before randomisation. SOFT^10^, ^11^ included only women who remained premenopausal after completion of neoadjuvant or adjuvant chemotherapy or women in whom adjuvant tamoxifen alone was considered suitable treatment. Of 1087 patients treated with chemotherapy in SOFT, 453 (41·7%) had received tamoxifen for an average of 4 months before study entry. In TEXT, adjuvant chemotherapy was optional and, if administered, was given concurrently with triptorelin. Tamoxifen or aromatase inhibition started after completion of chemotherapy. If chemotherapy was not given, tamoxifen or the aromatase inhibitor started 6–8 weeks after the initiation of triptorelin. The proportions of women who received chemotherapy were similar in SOFT (1060 [53·1%] of 1998 women), TEXT (1578 [59·9%] of 2635 women), and HOBOE (442 [62·9%] of 703 women). Use of adjuvant chemotherapy was not allowed in ABCSG XII; 97 [5·7%] of 1694 women in this trial had neoadjuvant chemotherapy before randomisation. Adjuvant trastuzumab was allowed in women with HER2-positive tumours in TEXT, SOFT, and HOBOE; ABCSG XII predated the use of trastuzumab and HER2 status was not ascertained.

Of 7030 women included in the analysis, 888 (12·6%) had a breast cancer recurrence, and 418 (5·9%) deaths occurred, of which 54 (12·9%) were from causes unrelated to breast cancer and without recorded disease recurrence. Overall median follow-up from the four trials was 8·0 years (IQR 6·1–9·3).

**Figure 1:**
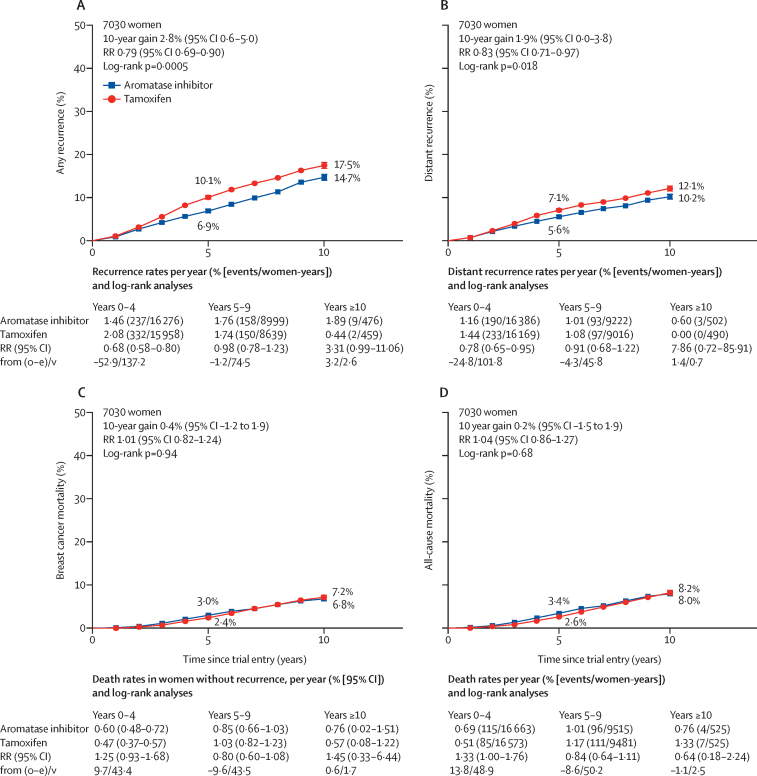
Aromatase inhibitors versus tamoxifen in premenopausal women (A) Any recurrence, (B) distant recurrence, (C) breast cancer mortality, and (D) all-cause mortality. O–E=observed minus expected. RR=rate ratio. V=variance of O–E.

**Figure 2:**
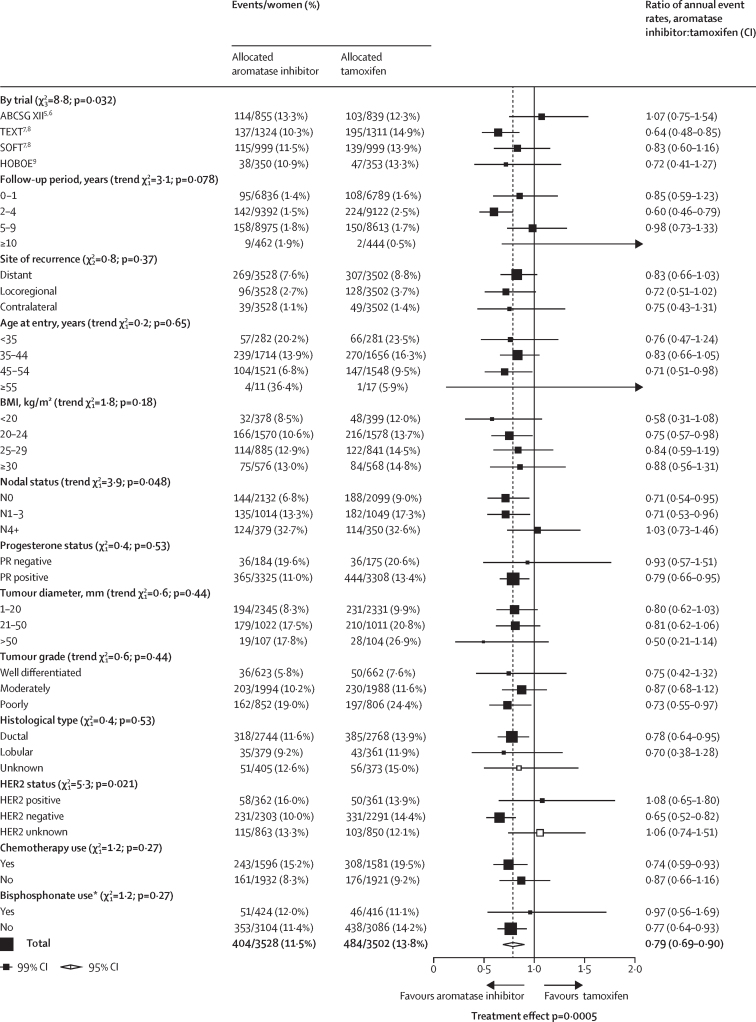
Subgroup analyses of any first recurrence (including locoregional recurrence, distant recurrence, and new contralateral disease) in premenopausal women treated with aromatase inhibitors or tamoxifen White squares represent women with unknown status within a subgroup. BMI=body-mass index. *Randomised bisphosphonate use.

Estimates of the 10-year risks of any recurrence, distant recurrence, breast cancer mortality, and all-cause mortality from combined analyses of the four trials are shown in figure 1. There was a reduction in the rate of any recurrence for women allocated to an aromatase inhibitor group compared with those assigned to tamoxifen (RR 0·79, 95% CI 0·69–0·90; p=0·0005). The main benefit from aromatase inhibitors on any recurrence was seen in years 0–4 of follow-up, the period when treatments differed, with a significant reduction in the rate of recurrence (RR 0·68, 99% CI 0·55–0·85; p<0·0001), with no further benefit, or loss of benefit, in years 5–9 (RR 0·98, 99% CI 0·73–1·33; p=0·89) or beyond year 10 (figure 2). The 5-year absolute risk of breast cancer recurrence was 3·2% (95% CI 1·8–4·5) lower in the aromatase inhibitor group than in the tamoxifen group (6·9% *vs* 10·1%, figure 1A), with a similar absolute difference in 10-year recurrence: 14·7% in the aromatase inhibitor group versus 17·5% in the tamoxifen group. Distant recurrence was reduced in the aromatase inhibitor group versus the tamoxifen group (RR 0·83, 95% CI 0·71–0·97; p=0·018; figure 1B, appendix p 4), but, with a median follow-up of 8·0 years, there was no significant difference in breast cancer mortality (1·01, 0·82–1·24; p=0·94; figure 1C) or all-cause mortality (1·04, 0·86–1·27; p=0·68; figure 1D, appendix p 6). The risk ratio for breast cancer mortality after being treated with an aromatase inhibitor compared with being treated with tamoxifen was 1·25 (99% CI 0·85–1·85) in years 0–4 of follow-up and 0·80 (0·54–1·19) in years 5–9 (figure 1C, appendix p 9).

Subgroup analyses of any recurrence, distant recurrence, and breast cancer mortality are shown in figure 2 and the appendix (pp 8–9). The proportional reductions in locoregional recurrence (RR 0·72, 95% CI 0·55–0·94; p=0·014; appendix p 3), and new contralateral disease (0·75, 0·50–1·15) as first event were of similar magnitude to the reductions in distant recurrence (figure 2, appendix pp 3, 5), so subgroup analyses were done for any recurrence (distant, local, and contralateral recurrence combined). None of these tests for heterogeneity in treatment efficacy by patient or tumour characteristics were significant for subgroup analyses (figure 2). The test for heterogeneity suggested slight differences between the four trial results for any recurrence; however, this was not statistically significant (p=0·032). The test for heterogeneity for nodal involvement was not significant (p=0·048); there was no apparent benefit from aromatase inhibitors over tamoxifen in N4+ disease (figure 2). There was weak evidence that HER2 status might affect the benefit of aromatase inhibitor over tamoxifen for recurrence (p=0·021, figure 2): recurrence rates were similar with aromatase inhibitors and tamoxifen in women with HER2-positive tumours (16·0% *vs* 13·9%), although just 723 (15·7%) of 4594 women with ER-positive status had HER2-positive disease, and hence there were few events and a wide CI. No additional benefit from aromatase inhibitor was apparent, either, in tumours with unknown HER2 status (RR 1·06, 99% CI 0·74–1·51); most of these patients were from ABCSG XII, which did not measure HER2 status. The proportional reduction in recurrence did not vary by other patient or tumour characteristics, including age, BMI, tumour size, tumour grade, or histological subtype. Analysis of any recurrence in women by ER and progesterone status is shown in the appendix (p 10).

The 10-year risks of recurrence by nodal status, progesterone status, and tumour grade are shown in appendix (pp 11–13). The proportional reductions observed in women with node-negative disease and in those with one to three positive nodes are identical (node -negative RR 0·71 [95% CI 0·57–0·89] *vs* N1–3 0·71 [0·58–0·91]), and the 5-year absolute benefits were larger in the higher risk N1–3 group than in the node-negative group: 4·8% versus 3·1%. However, there was no apparent improvement in the 5-year recurrence with an aromatase inhibitor compared with tamoxifen in the 729 (10·4%) of 7030 women with four or more positive nodes (RR 1·03 [95% CI 0·79–1·34]).

**Figure 3:**
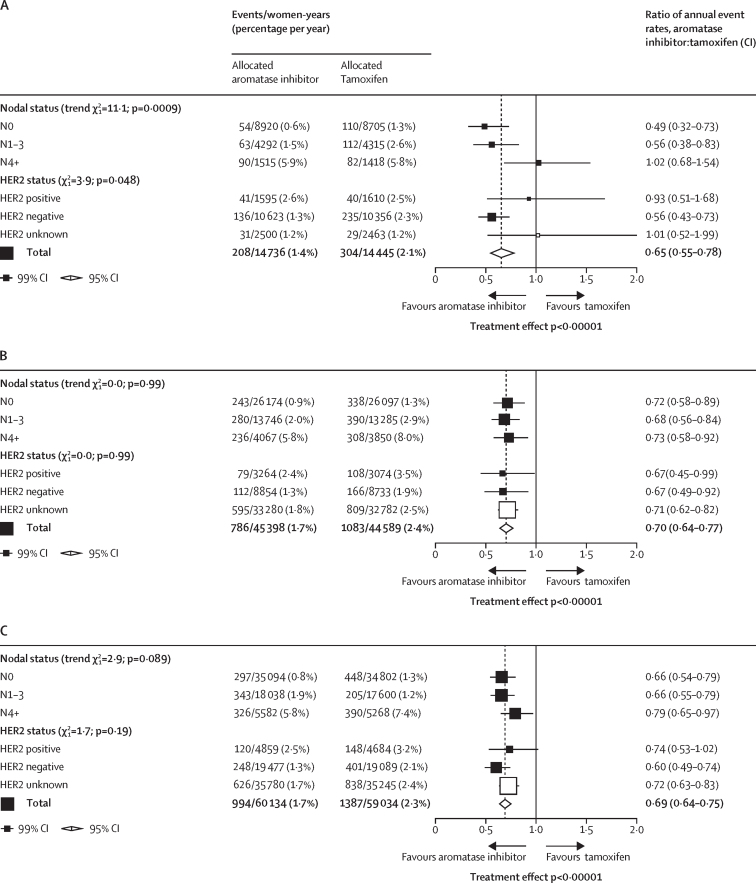
Post-hoc subgroup analyses of any recurrence aromatase inhibitors versus tamoxifen during the periods where treatments differed (A) Premenopausal women. (B) Postmenopausal women (published data from EBCTCG meta-analysis^2^ of aromatase inhibitor versus tamoxifen in early breast cancer). (C) All women. White squares represent women with unknown status within a subgroup.

To enhance statistical power to investigate differential efficacy within subgroups, we analysed aromatase inhibitor versus tamoxifen within nodal and HER2 status subsets only during the period when the treatments differed (post-hoc analysis; figure 3A). As most of the benefit from aromatase inhibitor was seen in this period, the reduction in any recurrence with aromatase inhibitor was larger and more highly significant. However, the recurrence reductions for N0 (RR 0·49) and N1–3 (0·56) tumours were similar, but no benefit was seen in N4+ (1·02), leading to a p value of 0·0009. To put this unexpected finding in perspective, we did a post-hoc analysis comparing the nodal status subgroup finding for premenopausal women with those we have previously reported for aromatase inhibitor versus tamoxifen in postmenopausal women,^2^ when there was no suggestion of any lesser benefit in N4+ disease (figure 3B). Similarly, the apparently lesser benefit from aromatase inhibitors in premenopausal women with HER2-positive compared with HER2-negative disease was not replicated in postmenopausal women. When data from premenopausal and postmenopausal women were combined, aromatase inhibitors were superior to tamoxifen in all nodal and HER2 status categories (figure 3C).

We repeated the subgroup analyses using data just during the period when treatments differed (appendix p 7), and the heterogeneity between trial results was still apparent (p=0·038). Few patients received chemotherapy in ABCSG XII compared with those in other trials, but proportional reductions in recurrence were not significantly greater in those receiving chemotherapy (appendix p 14); combining data from the other three trials, the reduction in recurrence with aromatase inhibitors compared with tamoxifen was larger in patients not receiving than those receiving chemotherapy: RR 0·64 (95% CI 0·46–0·89), p=0·0075, versus 0·74 (0·62–0·88), p=0·44; appendix p 15), respectively. Half of the women in ABCSG XII were allocated bisphosphonate but there was no greater benefit in the absence than presence of bisphosphonate (RR 0·97 [99% CI 0·56–1·69] *vs* 0·77 [99% CI 0·64–0·93]; p=0·29; figure 2, appendix p 14).

**Figure 4:**
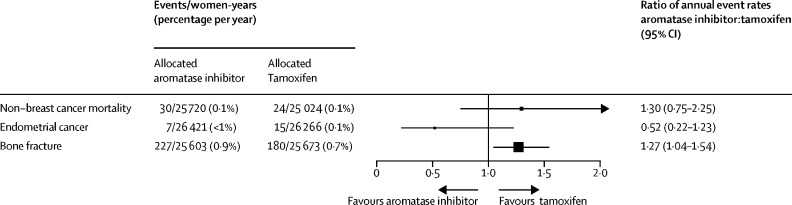
Non-breast cancer mortality, endometrial cancer incidence, and bone fracture incidence in trials of aromatase inhibitors versus tamoxifen in premenopausal women

Because the trials included only premenopausal women, few non-breast cancer deaths occurred: 30 (0·9%) of 3528 women in the aromatase inhibitor group versus 24 (0·7%) of 3502 women in the tamoxifen group died without breast cancer recurrence (RR 1·30, 95% CI 0·75–2·25; p=0·34; figure 4, appendix p 16). Most of these non-breast cancer deaths were due to second primary cancer: 22 in the aromatase inhibitor group versus ten in the tamoxifen group (p=0·015). These deaths were distributed across several second primary cancer sites: lung (six in the aromatase inhibitor group *vs* four in the tamoxifen group), ovarian (four *vs* one), pancreatic (four *vs* one), haematological (three *vs* one), and other specified sites (five *vs* three), and there was no difference in the overall incidence of fatal or non-fatal second cancers (RR 1·08, 95% CI 0·84–1·40; p=0·55; appendix p 16). Deaths from other cancers were unrelated to tumour size or nodal status (appendix p 17). The 5-year incidence of endometrial cancer (defined as any uterine cancer except cervix cancer) was also low: seven (0·2%) of 3528 women in the aromatase inhibitor group versus 15 (0·3%) of 3502 in the tamoxifen group (p=0·14, figure 4, appendix p 18). Individual patient data on bone fractures were available from ABCSG XII, SOFT, and TEXT; no fractures were recorded in HOBOE. When combining these data, there were more fractures among women allocated aromatase inhibitors than in those assigned to tamoxifen: 227 (6·4%) of 3528 versus 180 (5·1%) of 3502 (RR 1·27, 95% CI 1·04–1·54; p=0·017; figure 4, appendix p 18).

Toxicity from aromatase inhibitors and tamoxifen in premenopausal women, as reported from the individual trial publications, was similar to that seen in postmenopausal women. Higher rates of osteoporosis were reported in those taking aromatase inhibitors compared with those taking tamoxifen, whereas endometrial abnormalities, including uterine polyps and endometrial cancers, were more frequent in those receiving tamoxifen (appendix pp 19–20).

## Discussion

This meta-analysis of 7030 premenopausal women receiving ovarian function supression for early-stage ER-positive breast cancer in four randomised trials showed a reduction in recurrence rates favouring aromatase inhibitor over tamoxifen. For the patient population in these trials, in which 4231 (60·2%) of 7030 women had node-negative cancer, there was an absolute reduction of about 3% in 5-year and 10-year recurrence risk. As in the previous EBCTCG meta-analysis^2^ of aromatase inhibitors versus tamoxifen in postmenopausal women, almost all the benefit of aromatase inhibitors over tamoxifen was observed in the period when treatments differed, with no further gain or loss of benefit after year 5.

Although this meta-analysis found no difference in breast cancer or all-cause mortality between the two treatment groups, this lack of difference might be explained by the limited duration of follow-up (median follow up 8·0 years). Given the significant reduction in distant recurrence with aromatase inhibitors (p=0·018), it would be premature to conclude that survival is not improved with aromatase inhibitors. A meta-analysis of trials comparing adjuvant aromatase inhibitors versus tamoxifen therapy in postmenopausal women showed no significant survival advantage with a median follow-up of 5 years.^16^ However, a second meta-analysis with longer follow-up did establish a reduction in breast cancer mortality and all-cause mortality.^2^ This late benefit highlights the importance of long-term follow-up of breast cancer trials, particularly for women with hormone receptor-positive disease whose risk of recurrence persists at about the same annual rate for up to 20 years after diagnosis.^17^

Although the overall reduction in recurrences with aromatase inhibitors compared with tamoxifen was highly significant, results of individual trials were inconsistent; three showed benefit from aromatase inhibitors over tamoxifen, whereas no reduction in recurrence was apparent in the ABCSG XII trial. The shorter, 3-year duration of endocrine treatment in ABCSG XII did not explain the heterogeneity between the four trial results, since the difference between trials persisted in analyses limited to the period when treatments differed. The use of different aromatase inhibitor drugs is also unlikely to explain the differences in trial results, as no differences were seen between drugs in the EBCTCG meta-analysis of aromatase inhibitors versus tamoxifen in postmenopausal women,^2^ or in direct comparisons between treatments.^18^, ^19^, ^20^, ^21^ Patients in ABCSG XII were at somewhat lower risk than those in other trials, with 1212 (67·2%) of 1803 women node negative, but the proportional reductions in recurrence with aromatase inhibitor compared with tamoxifen were at least as big in node-negative as in node-positive disease, so this also cannot be the reason for the lesser benefit from aromatase inhibitors in the ABCSG XII trial. Another possible factor is that ovarian function supression was achieved with goserelin in ABCSG XII and triptorelin in other trials, but we are not aware of any data indicating that these drugs are any more or less efficacious in achieving ovarian function supression. There were fewer high-risk patients in ABCSG XII and few received chemotherapy (97 [5·7%] of 1694), but subgroup analyses suggest that these differences should not have much affected the proportional reductions in recurrence. So, the inconsistent trial results are most likely a chance finding: the p-value for heterogeneity (p=0·032) did not reach the 2p of less than 0·01 threshold for significance in subgroup investigations.

Subgroup analyses also suggested greater benefit from aromatase inhibitors compared with tamoxifen for any recurrence in HER2-negative disease than in HER2-positive disease (RR 0·65 *vs* 1·08, p=0·021). However, the HER2-positive subgroup included a small number of women and the difference between HER2-positive and negative tumours also did not reach the 2p of less than 0·01 level of significance prespecified for subgroup analyses. Additionally, the EBCTCG meta-analysis^2^ of aromatase inhibitors versus tamoxifen in postmenopausal women found similar proportional reductions in recurrence in HER2-positive and HER2-negative disease so the apparent lack of benefit from aromatase inhibitors over tamoxifen in HER2-positive tumours could also be a chance finding.

The most unexpected subgroup finding was the apparent lack of superiority of aromatase inhibitors over tamoxifen in women with four or more involved nodes. This difference was highly significant in subgroup analyses limited to the period when treatments differed. By contrast, in postmenopausal women, aromatase inhibitors appeared just as effective as tamoxifen when four or more nodes were involved as in those with fewer nodes involved. Moreover, the proportional reductions in recurrence have not differed by nodal status in any previous meta-analysis of endocrine therapies. So, despite the highly significant heterogeneity, this could be another chance finding, particularly given that 13 separate subgroup investigations were done. Splitting the overall 12·1 χ^2^ treatment effect across multiple subgroups can lead to statistically unreliable findings.^14^ With no good previous reasons for anticipating that the proportional effects of treatment might be so different in these different nodal status categories, the proportional risk reduction that is suggested by the overall results of the meta-analysis (RR 0·79) might provide a better guide to the proportional risk reductions that would be achieved in different nodal subgroups than the apparent results in each subgroup.^14^ If so, those patients at highest recurrence risk, as defined by clinicopathological characteristics, should derive the greatest benefit from aromatase inhibitors, as is usually assumed.^22^ There were, however, too few patients in some subgroups, for example those with PR-negative or lobular tumours, for meaningful assessment of any potential differential benefit.

Ovarian function supression might be less effective at fully supressing ovarian oestrogen production in women younger than 35 years of age,^23^ who have a higher risk of recurrence than older women.^24^, ^25^, ^26^ However, our subgroup analysis of recurrence by age showed no significant trend across the age groupings. Subgroup analyses of the ABCSG XII trial suggested that women with a BMI of 25kg/m^2^ or higher treated with anastrozole plus goserelin had worse disease-free survival and overall survival than those treated with tamoxifen plus goserelin.^9^, ^27^ However, our analyses showed similar recurrence reductions with aromatase inhibitors compared with tamoxifen across BMI groupings.

Individual patient level data on quality of life was not available for this meta-analysis. However, a combined analysis of patient-reported outcomes in TEXT and SOFT,^15^ found no strong indication to favour either exemestane plus ovarian function supression or tamoxifen plus ovarian function supression with respect to overall quality of life.

In summary, results from this meta-analysis suggest that using an aromatase inhibitor rather than tamoxifen in addition to ovarian function supression for premenopausal women reduces the absolute risk of recurrence by 3% at 5 and 10 years. Reassuringly, we found no increase in non-breast cancer deaths over the 10-year follow-up period. Deaths from other cancers were unrelated to tumour size or nodal status, so are unlikely to be misclassified breast cancer metastases. Such events are rare in these younger women, as are the other known side-effects of bone fracture with aromatase inhibitors and endometrial cancer with tamoxifen. Some of these side-effects can be mitigated, for example by the use of bisphosphonates to preserve bone density and reduce bone fractures in women receiving aromatase inhibitors.^28^ The effects of endocrine therapy and ovarian function supression on quality of life also need to be carefully considered alongside the expected improvement in disease outcomes demonstrated in this meta-analysis.

Correspondence to: EBCTCG Secretariat, Clinical Trial Service Unit, Nuffield Department of Population Health, Richard Doll Building, Oxford OX3 7LF, UK **bc.overview@ctsu.ox.ac.uk**

## Data sharing

The data sharing policy is available online at https://www.ndph.ox.ac.uk/data-access.

## Declaration of interests

RG and RKH report that EBCTCG is supported by a Cancer Research UK grant paid to the University of Oxford. JBe reports institutional grants or contracts from Amgen, AstraZeneca, Bayer, Merck, Pfizer, Roche, and Sanofi-Aventis; and payment from UpToDate for a chapter on breast cancer prediction. SS reports institutional grants or contracts from Kailos Genetics, Genentech/Roche, and Breast Cancer Research Foundation; consulting fees from Molecular Templates, Silverback Therapeutics, Genentech/Roche, Athenex, Lilly Pharmaceuticals, Merck, Exact sciences, Daiichi-Sankyo, AstraZeneca, Natera, Biotheranostics, and Bejing Medical Foundation; payments for non-promotional speaking from Daiichi Sankyo and Genentech/Roche; and support for attending meetings, travel, or both from Genentech/Roche and Caris. SS also reports participation on a Data Safety Monitoring Board or Advisory Board for AstraZeneca/BIG (Olympia trial); leadership or fiduciary roles on the National Surgical Adjuvant Breast and Bowel Project Board and Conquer Cancer Foundation Board; third party medical writing from Genentech/Roche; and other financial or non-financial interests, in Scientific Advisory Board for Inivata. PF reports support for travel overseas to lecture on SOFT and TEXT trials from Novartis and Ipsen. MG reports consulting fees from DaiichiSankyo, EliLilly, and Lifebrain; payments or honoraria for lectures, presentations, speakers bureaus, manuscript writing, or educational events from Amgen, AstraZeneca, Novartis, and PierreFabre; payment for expert testimony from Veracyte; and other financial or non-financial interests from Sandoz (an immediate family member is employed by the company). FP reports institutional grants or contracts from Roche, AstraZeneca, Pfizer, Merck Sharp & Dohme, Bayer, Incyte, Taiho Oncology, Janssen Cilag, Exelixis, Aileron, and Daiichi Sankyo; payments or honoraria for educational activities or advice on regulatory activities from Incyte, GlaxoSmithKline, Eli Lilly, Ipsen, Astellas, AstraZeneca, Roche, Bristol Myers Squibb, Bayer, Clovis, and Pierre Fabre; and a leadership or fiduciary role in the Italian Society of Medical Oncology. MMR reports support for IBCSG for SOFT and TEXT from Pfizer, Ipsen, TerSera, DebioPharm, and BCRF; grants or contracts to IBCSG from Novartis, Pfizer, Merck, Roche, AstraZeneca, and Bristol Myers Squibb; institutional grants or contracts from Bristol Myers Squibb and Bayer; consulting fees from Tolmar; and payments or honoraria for lectures, presentations, speakers bureaus, manuscript writing, or educational events from Bristol Myers Squibb and WebMD. MMR also reports participation on a Data Safety Monitoring Board or Advisory Board for ABCSG; and a leadership or fiduciary role in IBCSG. All other authors declare no competing interests.
