# Correction to *Lancet Oncol* 2022; 23: 382–92

**DOI:** 10.1016/S1470-2045(22)00132-2

**Published:** 2022-04

*Early Breast Cancer Trialists’ Collaborative Group (EBCTCG). Aromatase inhibitors versus tamoxifen in premenopausal women with oestrogen receptor-positive early-stage breast cancer treated with ovarian suppression: a patient-level meta-analysis of 7030 women from four randomised trials.* Lancet Oncol *2022; **23:** 382–92*—In this Article, in the table, surgery type for the ABCSG XII trial should read “18% mastectomy; 80% partial mastectomy; 2% unknown”, and the y-label on figure 1C was incorrect. These corrections has been made to the online version as of March 28, 2022.

