# Supplementary material for: Aromatase inhibitors versus tamoxifen in premenopausal women with oestrogen receptor-positive early-stage breast cancer treated with ovarian suppression: a patient-level meta-analysis of 7030 women from four randomised trials
Source: Lancet Oncol. 2022 Mar;23(3):382–92. doi: 10.1016/S1470-2045(21)00758-0 (PMC8885431; doi:10.1016/S1470-2045(21)00758-0)
Supplement: Supplementary appendix [file mmc1.pdf]

THE LANCET
Oncology

Supplementary appendix

This appendix formed part of the original submission and has been peer reviewed. We post it as supplied by the authors.

Supplement to: Early Breast Cancer Trialists' Collaborative Group (EBCTCG). Aromatase inhibitors versus tamoxifen in premenopausal women with oestrogen receptor-positive early-stage breast cancer treated with ovarian suppression: a patient-level meta-analysis of 7030 women from four randomised trials. *Lancet Oncol* 2022; published online Feb 3. https://doi.org/10.1016/S1470-2045(21)00758-0.

P1: Supplementary appendix for:

Aromatase inhibitors versus tamoxifen in premenopausal women with oestrogen receptor-positive early-stage breast cancer treated with ovarian suppression: a patient-level meta-analysis of 7030 women from four randomised trials

P2: Webappendix: Supplementary figures and tables for "Aromatase inhibitors versus tamoxifen in premenopausal women with oestrogen receptor-positive early-stage breast cancer treated with ovarian suppression: a patient-level meta-analysis of 7030 women from four randomised trials"

CONTENTS LIST (Click on any item with a page number to jump to it)

Page 3: Kaplan-Meier graphs, for 4 different endpoints (any recurrence, distant recurrence at any time, local recurrence as first event, contralateral recurrence as first event)

Pages 4-6: Forest plots, one line per-trial for 8 different endpoints (any recurrence, distant recurrence at any time, local recurrence as first event, contralateral recurrence as first event, breast cancer mortality, death without recurrence, all-cause mortality)

Page 7: Subgroup analyses for any recurrence; only data during periods when treatments differed

Page 8: Subgroup analyses for distant recurrence

Page 9: Subgroup analyses for breast cancer mortality

Page 10: Subgroup analysis for any recurrence split by ER and PR

Pages 11-13: 10-year risk of recurrence within subgroups (PR status, nodal status and tumour grade)

Page 14: Sensitivity analysis any recurrence by chemotherapy use and bisphosphonate use

Page 15: Sensitivity analysis any recurrence by chemotherapy use excluding ABCSG XII

Page 16: Mortality by cause & incidence of second cancers and bone fractures

Page 17: Death without recurrence by tumour size and nodal status

Page 18: Kaplan-Meier graphs for toxicity (death without recurrence, endometrial cancer incidence, bone fracture incidence)

Pages 19-20: Toxicity (as reported in trials of aromatase inhibitor versus tamoxifen in premenopausal women)

Pages 21-29: Statistical Analysis Plan including list of relevant trials and publications, and variables requested

P3: 5 year cumulative risk of (A) any recurrence, (B) distant recurrence at any time, (C) isolated local recurrence as first event, (D) contralateral recurrence as first event in trials of AI versus tamoxifen in premenopausal women

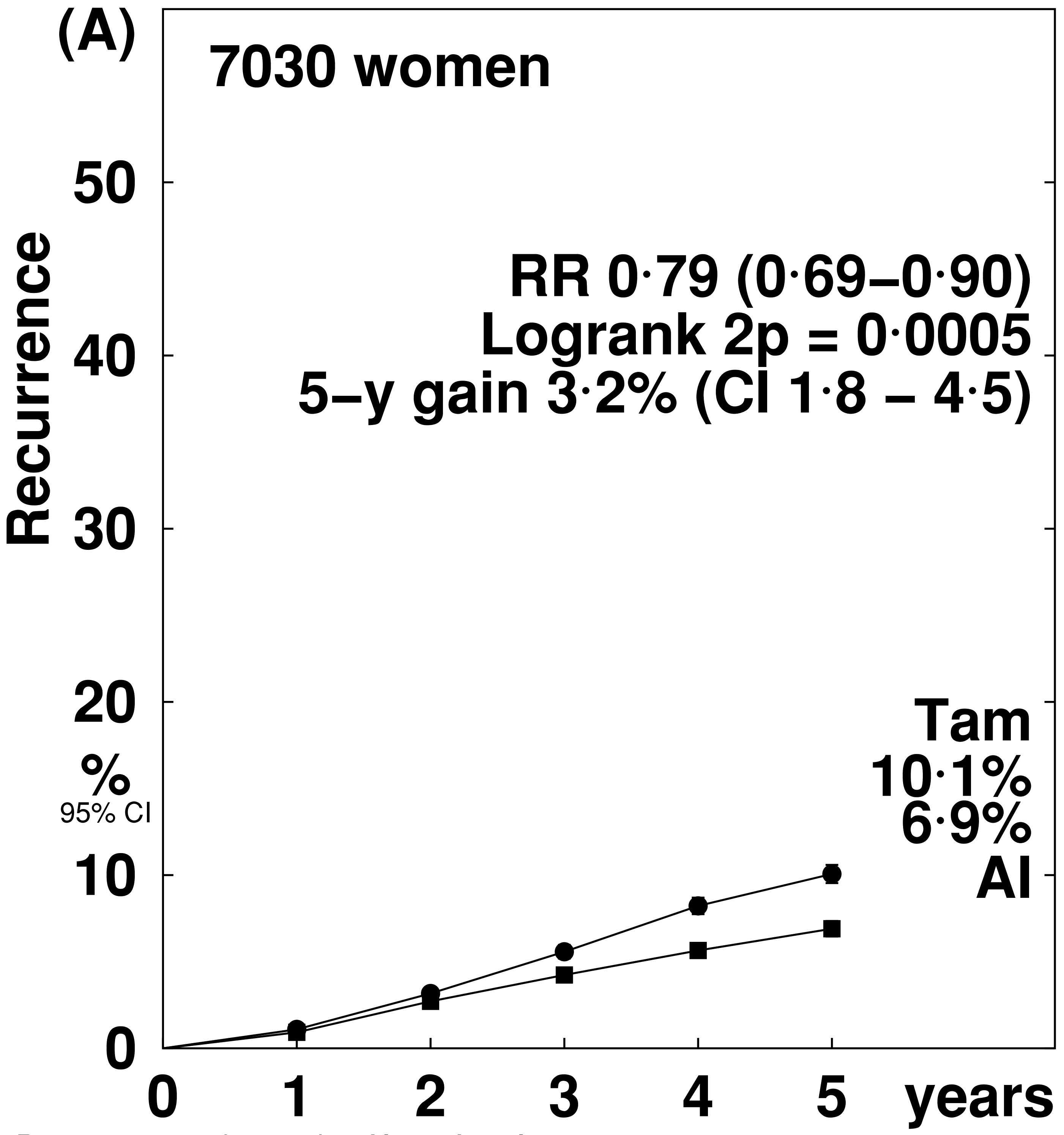

Recurrence rates (% / year) and logrank analyses

Allocation	Years 0 – 1	Years 2 – 4	Year 5+
AI	1·39 (95 / 6854)	1·51 (142 / 9421)	1·76 (167 / 9476)
Tam	1·59 (108 / 6805)	2·45 (224 / 9152)	1·67 (152 / 9098)
Rate ratio, from	0·85 CI 0·65 – 1·13	0·60 CI 0·49 – 0·74	1·03 CI 0·82 – 1·28
(O–E) / V	–7·7 / 49·0	–45·2 / 88·2	1·9 / 77·1

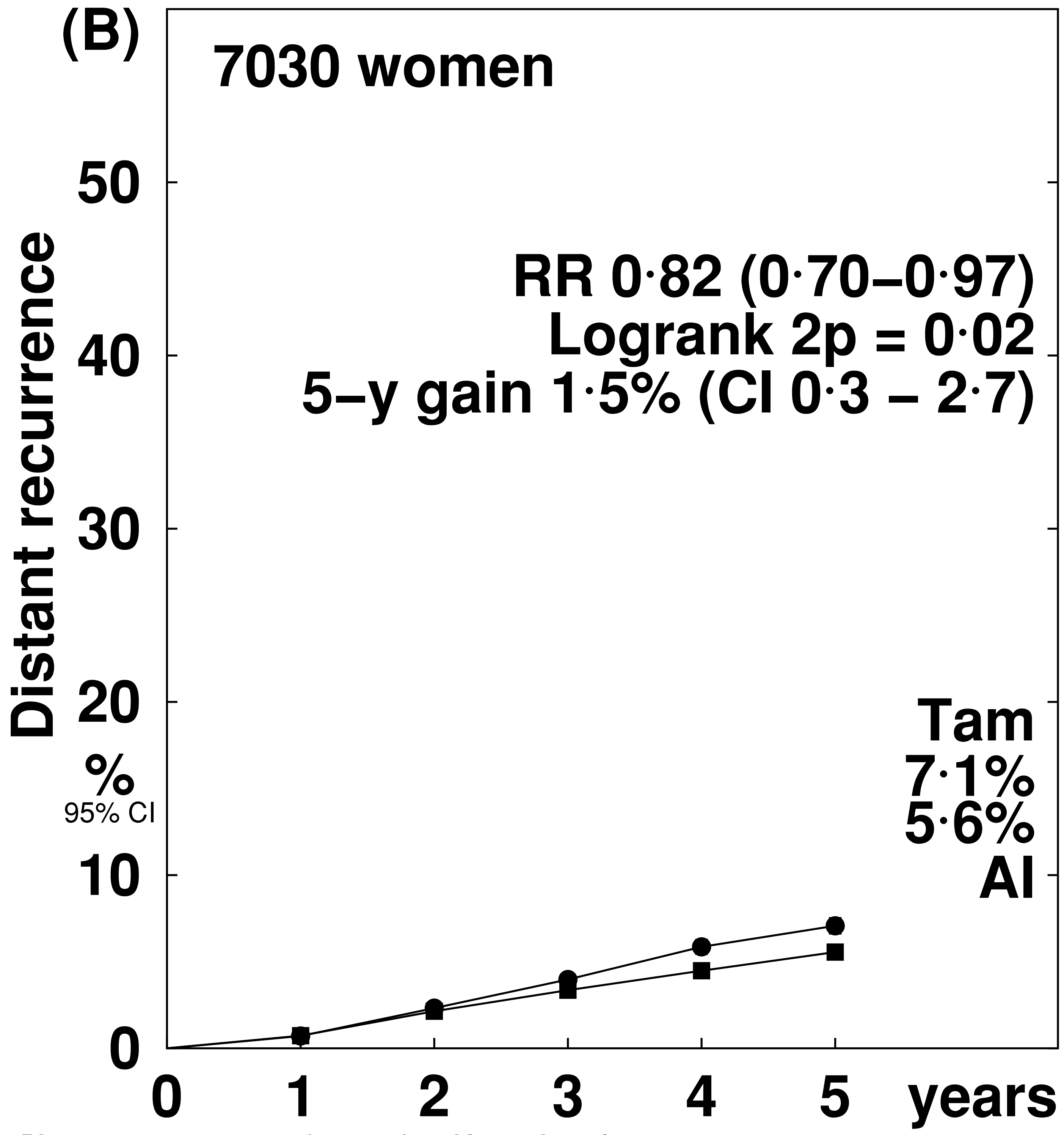

Distant recurrence rates (% / year) and logrank analyses

Allocation	Years 0 – 1	Years 2 – 4	Year 5+
AI	1·09 (75 / 6870)	1·21 (115 / 9516)	0·99 (96 / 9724)
Tam	1·16 (79 / 6831)	1·65 (154 / 9337)	1·02 (97 / 9507)
Rate ratio, from	0·92 CI 0·67 – 1·27	0·71 CI 0·56 – 0·91	0·92 CI 0·69 – 1·23
(O–E) / V	–3·0 / 37·2	–22·1 / 64·8	–3·8 / 46·8

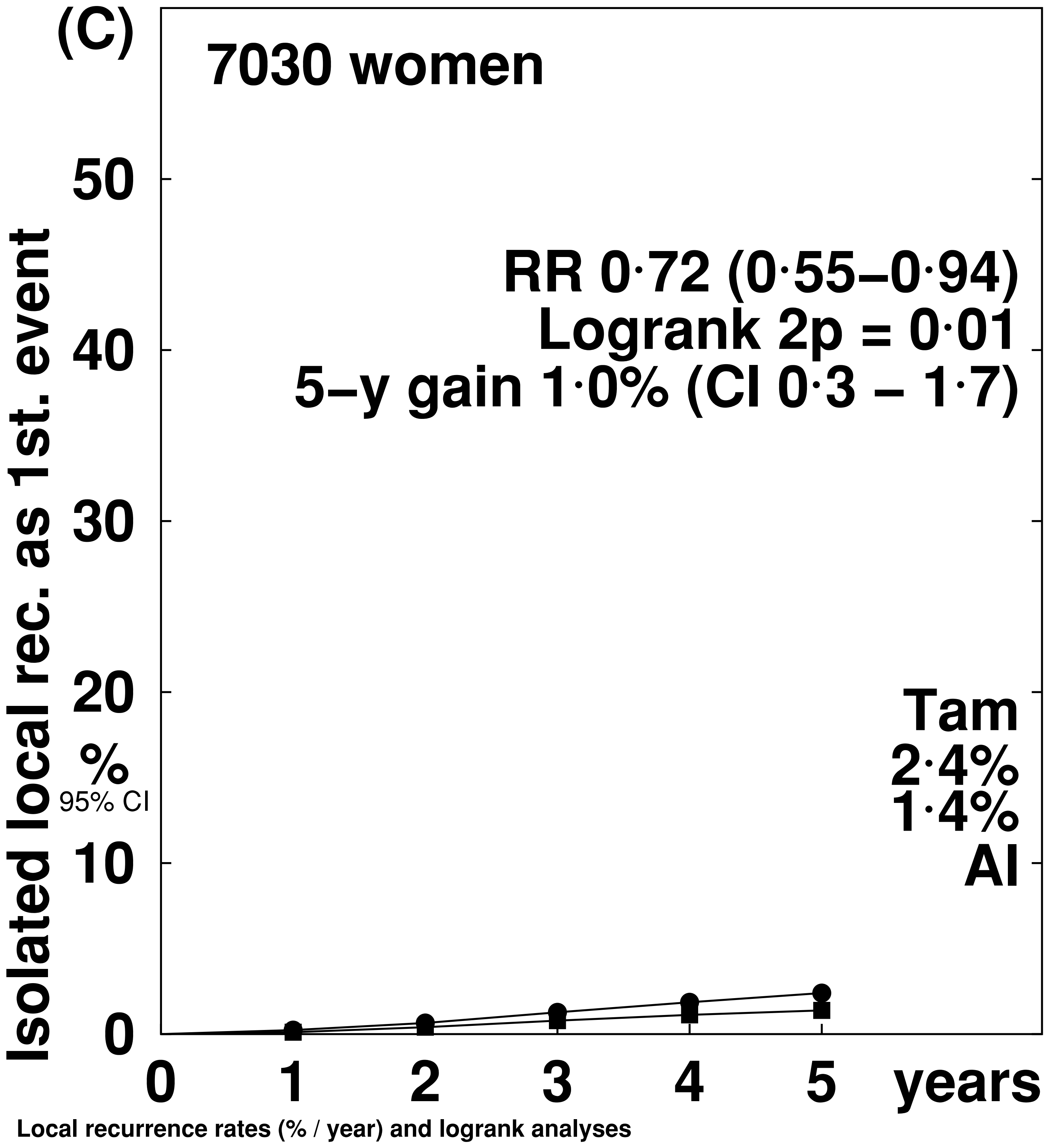

Local recurrence rates (% / year) and logrank analyses

Allocation	Years 0 – 1	Years 2 – 4	Year 5+
AI	0·20 (14 / 6854)	0·34 (32 / 9421)	0·53 (50 / 9476)
Tam	0·32 (22 / 6805)	0·59 (54 / 9152)	0·57 (52 / 9098)
Rate ratio, from	0·62 CI 0·32 – 1·20	0·57 CI 0·37 – 0·87	0·92 CI 0·62 – 1·37
(O–E) / V	–4·2 / 8·9	–12·1 / 21·3	–2·0 / 25·0

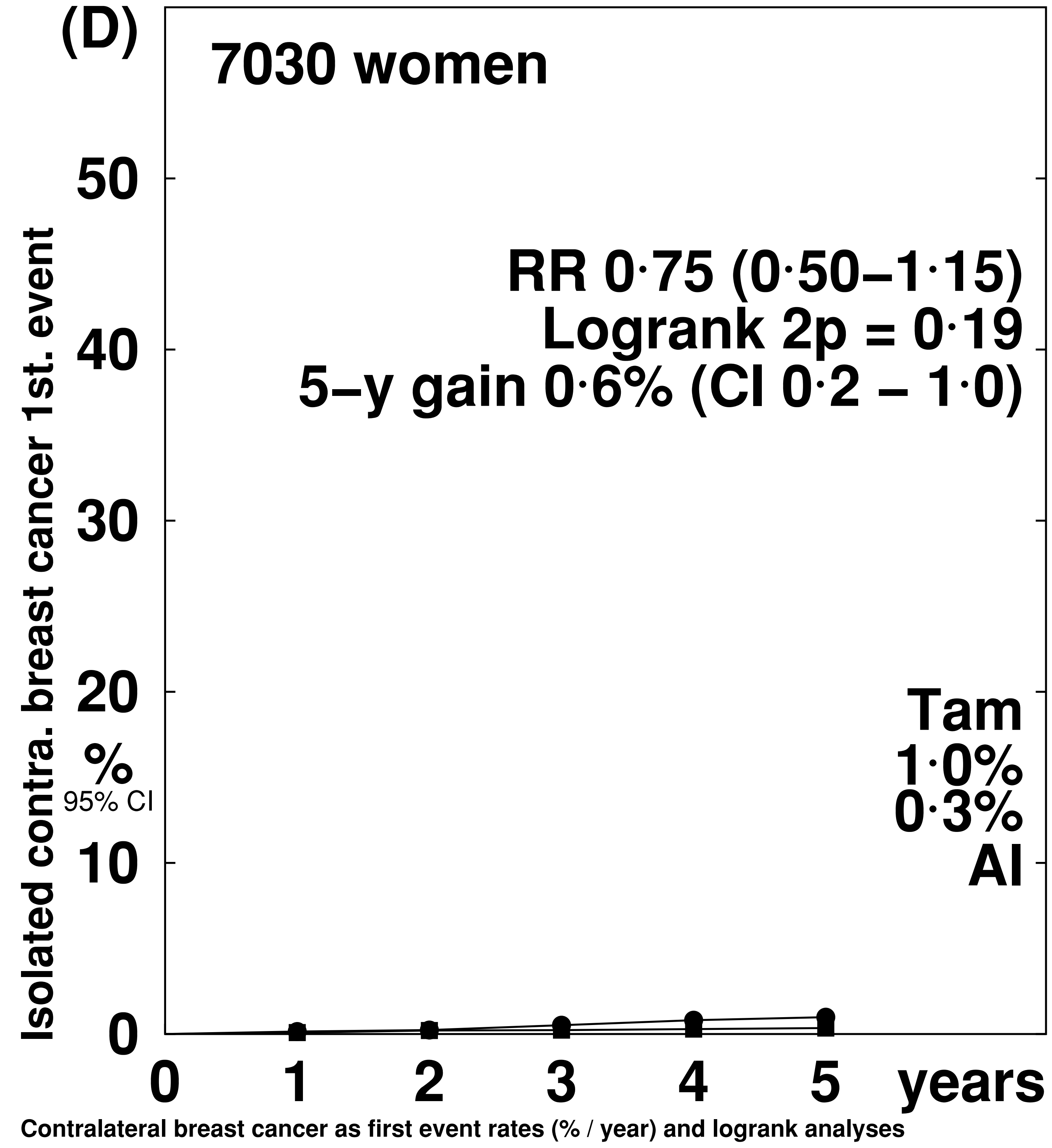

Contralateral breast cancer as first event rates (% / year) and logrank analyses

Allocation	Years 0 – 1	Years 2 – 4	Year 5+
AI	0·10 (7 / 6854)	0·05 (5 / 9421)	0·28 (27 / 9476)
Tam	0·12 (8 / 6805)	0·25 (23 / 9152)	0·20 (18 / 9098)
Rate ratio, from	0·87 CI 0·32 – 2·41	0·25 CI 0·12 – 0·54	1·41 CI 0·78 – 2·53
(O–E) / V	–0·5 / 3·7	–9·5 / 6·9	3·8 / 11·2

P4: Recurrence and distant recurrence at any time in trials testings AI versus tamoxifen in premenopausal women

Recurrence

Year code and study name	Treatment comparison	Events/Women: Allocated AI	Events/Women: Allocated Tam	AI events: Logrank O−E	AI events: Variance of O−E
**AI vs tamoxifen (premenopausal women with ovarian suppression)**					
99B1+2 Austrian BCSG XII	Gos;(Ana vs Tam)±Zol 3yr	114/855	103/839	2·9	52·3
03< TEXT / IBCSG 25−02	Trip;(Exe vs Tam) 5yr	137/1324	195/1311	−35·9	80·4
03E SOFT / IBCSG 24−02	Trip;(Exe vs Tam) 5yr	115/999	139/999	−11·2	61·1
04]1+5 IT Naples HOBOE	Trip;(Let vs Tam) 5yr	38/350	47/353	−6·8	20·5
**Total**		**404/ 3528 (11·5%)**	**484/ 3502 (13·8%)**	**−51·0**	**214·3**

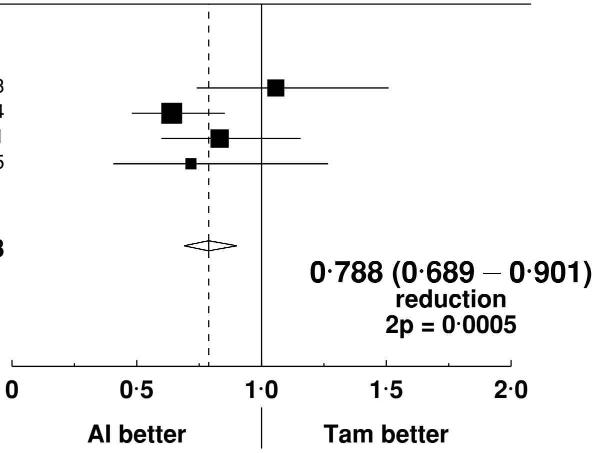

99% or 95% confidence intervals

Heterogeneity between 4 trials: χ^2_3 = 8·4; p = 0·04

Treatment effect 2p = 0·0005

Distant recurrence at any time

Year code and study name	Treatment comparison	Events/Women: Allocated AI	Events/Women: Allocated Tam	AI events: Logrank O−E	AI events: Variance of O−E
**AI vs tamoxifen (premenopausal women with ovarian suppression)**					
99B1256 Austrian BCSG XII	Gos;(Ana vs Tam)±Zol 3yr	67/855	57/839	4·7	29·6
03< TEXT / IBCSG 25−02	Trip;(Exe vs Tam) 5yr	106/1324	135/1311	−19·1	58·3
03E SOFT / IBCSG 24−02	Trip;(Exe vs Tam) 5yr	85/999	103/999	−8·0	45·2
04]1+5 IT Naples HOBOE	Trip;(Let vs Tam) 5yr	28/350	35/353	−5·4	15·1
**Total**		**286/ 3528 (8·1%)**	**330/ 3502 (9·4%)**	**−27·8**	**148·3**

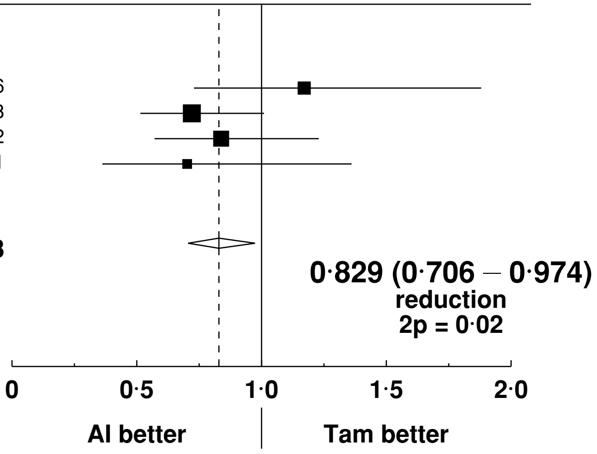

99% or 95% confidence intervals

Heterogeneity between 4 trials: χ^2_3 = 5·1; p > 0·1; NS

Treatment effect 2p = 0·02

P5: Local recurrence and first event and contralateral recurrence as first event in trials testings AI versus tamoxifen in premenopausal women

Local recurrence as first event

Year code and study name	Treatment comparison	Events/Women Allocated AI	Events/Women Allocated Tam	AI events Logrank O–E	AI events Variance of O–E
**AI vs tamoxifen (premenopausal women with ovarian suppression)**					
99B1+2 Austrian BCSG XII	Gos;(Ana vs Tam)±Zol 3yr	37/855	37/839	−0·8	18·1
03< TEXT / IBCSG 25−02	Trip;(Exe vs Tam) 5yr	29/1324	53/1311	−13·4	20·3
03E SOFT / IBCSG 24−02	Trip;(Exe vs Tam) 5yr	24/999	30/999	−2·7	13·4
04]1+5 IT Naples HOBOE	Trip;(Let vs Tam) 5yr	6/350	8/353	−1·3	3·5
**Total**		**96/ 3528 (2·7%)**	**128/ 3502 (3·7%)**	**−18·2**	**55·2**

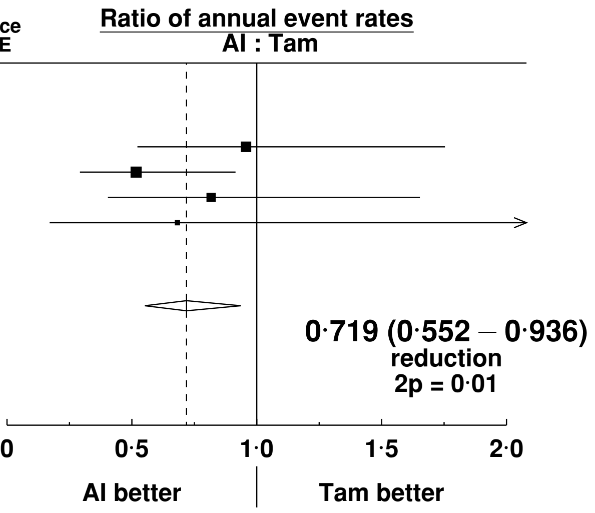

99% or 95% confidence intervals

Heterogeneity between 4 trials: $\chi^2_3 = 3{\cdot}9$; p > 0·1; NS

Treatment effect 2p = 0·01

Contralateral recurrence as first event

Year code and study name	Treatment comparison	Events/Women Allocated AI	Events/Women Allocated Tam	AI events Logrank O–E	AI events Variance of O–E
**AI vs tamoxifen (premenopausal women with ovarian suppression)**					
99B1+2 Austrian BCSG XII	Gos;(Ana vs Tam)±Zol 3yr	11/855	13/839	−1·3	6·0
03< TEXT / IBCSG 25−02	Trip;(Exe vs Tam) 5yr	11/1324	18/1311	−4·0	7·2
03E SOFT / IBCSG 24−02	Trip;(Exe vs Tam) 5yr	12/999	14/999	−1·3	6·4
04]1+5 IT Naples HOBOE	Trip;(Let vs Tam) 5yr	5/350	4/353	0·4	2·2
**Total**		**39/ 3528 (1·1%)**	**49/ 3502 (1·4%)**	**−6·2**	**21·8**

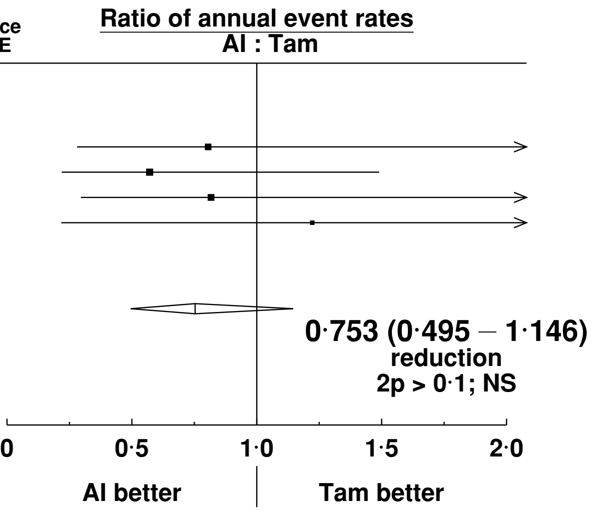

99% or 95% confidence intervals

Heterogeneity between 4 trials: $\chi^2_3 = 1{\cdot}1$; p > 0·1; NS

Treatment effect 2p > 0·1; NS

P6: Breast cancer mortality, death without recurrence and all-cause mortality in trials testings AI versus tamoxifen in premenopausal women

Breast cancer mortality

Year code and study name	Treatment comparison	Deaths/Women Allocated AI	Deaths/Women Allocated Tam	AI deaths Logrank O−E	AI deaths Variance of O−E
**AI vs tamoxifen (premenopausal women with ovarian suppression)**					
99B1+2 Austrian BCSG XII	Gos;(Ana vs Tam)±Zol 3yr	46/855	30/839	6·5	18·4
03< TEXT / IBCSG 25−02	Trip;(Exe vs Tam) 5yr	67/1324	83/1311	−9·2	36·7
03E SOFT / IBCSG 24−02	Trip;(Exe vs Tam) 5yr	63/999	52/999	6·6	27·9
04]1+5 IT Naples HOBOE	Trip;(Let vs Tam) 5yr	9/350	14/353	−3·2	5·6
**Total**		**185/ 3528 (5·2%)**	**179/ 3502 (5·1%)**	**0·7**	**88·6**

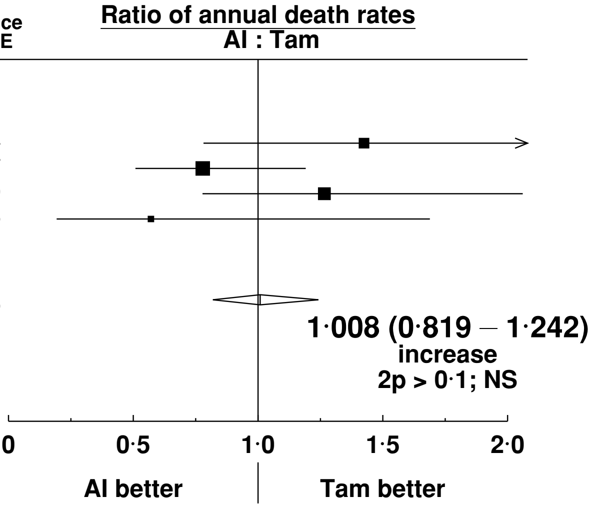

99% or 95% confidence intervals

Heterogeneity between 4 trials: $\chi^2_3 = 7{\cdot}9$; p = 0·05

Treatment effect 2p > 0·1; NS, adverse

Death without recurrence

Year code and study name	Treatment comparison	Deaths/Women Allocated AI	Deaths/Women Allocated Tam	AI deaths Logrank O−E	AI deaths Variance of O−E
**AI vs tamoxifen (premenopausal women with ovarian suppression)**					
99B1+2 Austrian BCSG XII	Gos;(Ana vs Tam)±Zol 3yr	8/855	4/839	2·1	3·0
03< TEXT / IBCSG 25−02	Trip;(Exe vs Tam) 5yr	12/1324	14/1311	−1·6	6·5
03E SOFT / IBCSG 24−02	Trip;(Exe vs Tam) 5yr	9/999	3/999	3·4	2·7
04]1+5 IT Naples HOBOE	Trip;(Let vs Tam) 5yr	1/350	3/353	−0·6	0·7
**Total**		**30/ 3528 (0·9%)**	**24/ 3502 (0·7%)**	**3·4**	**12·9**

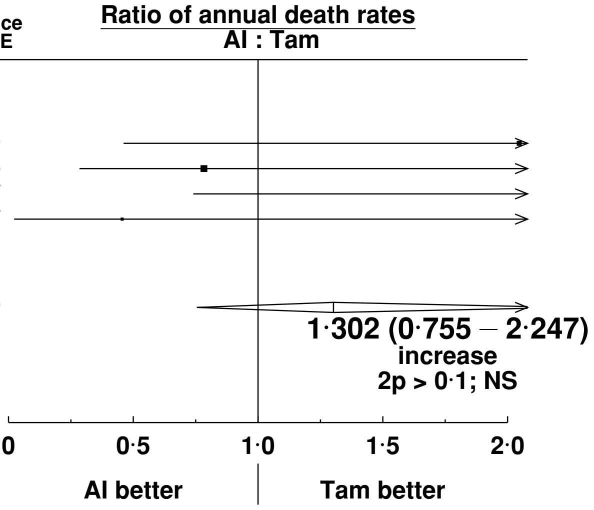

99% or 95% confidence intervals

Heterogeneity between 4 trials: $\chi^2_3 = 5{\cdot}8$; p > 0·1; NS

Treatment effect 2p > 0·1; NS, adverse

All-cause mortality

Year code and study name	Treatment comparison	Deaths/Women Allocated AI	Deaths/Women Allocated Tam	AI deaths Logrank O−E	AI deaths Variance of O−E
**AI vs tamoxifen (premenopausal women with ovarian suppression)**					
99B1+2 Austrian BCSG XII	Gos;(Ana vs Tam)±Zol 3yr	54/855	34/839	8·6	21·4
03< TEXT / IBCSG 25−02	Trip;(Exe vs Tam) 5yr	79/1324	97/1311	−10·8	43·1
03E SOFT / IBCSG 24−02	Trip;(Exe vs Tam) 5yr	72/999	55/999	10·0	30·7
04]1+5 IT Naples HOBOE	Trip;(Let vs Tam) 5yr	10/350	17/353	−3·7	6·4
**Total**		**215/ 3528 (6·1%)**	**203/ 3502 (5·8%)**	**4·2**	**101·5**

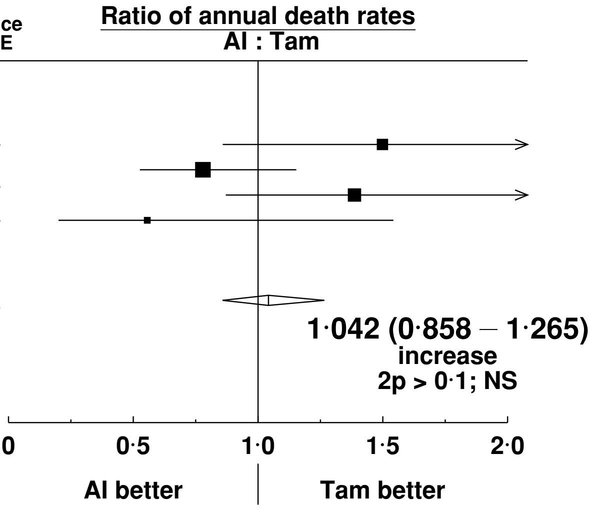

99% or 95% confidence intervals

Heterogeneity between 4 trials: $\chi^2_3 = 11{\cdot}5$; p = 0·009

Treatment effect 2p > 0·1; NS, adverse

P7: Subgroup analyses of AI vs tamoxifen in premenopausal women; any recurrence only during the time period when treatment differ

Category	Events/Women Allocated AI	Events/Women Allocated Tam	AI events Logrank O–E	AI events Variance of O–E	Ratio (& CI)
**(a) By trial** (χ^2_3 = 8.1; p = 0.04)					
ABCSG XII	31/855 (3·6%)	29/839 (3·5%)	0·2	14·5	
TEXT	76/1324 (5·7%)	130/1311 (9·9%)	−30·2	49·8	0·55 (0·38 – 0·79)
SOFT	84/999 (8·4%)	108/999 (10·8%)	−11·6	45·9	0·78 (0·53 – 1·14)
HOBOE	17/350 (4·9%)	37/353 (10·5%)	−11·0	13·1	0·43 (0·21 – 0·88)
**(b) Site of recurrence** (χ^2_2 = 4.5; 2p > 0.1; NS)					
Distant	159/3528 (4·5%)	208/3502 (5·9%)	−27·6	88·4	0·73 (0·56 – 0·96)
Isolated local	39/3528 (1·1%)	70/3502 (2·0%)	−16·5	27·0	0·54 (0·33 – 0·89)
Contralateral	10/3528 (0·3%)	26/3502 (0·7%)	−8·4	8·9	0·39 (0·16 – 0·92)
**(c) Age at entry** (trend χ^2_1 = 1.3; 2p > 0.1; NS)					
Age <35	35/282 (12·4%)	46/281 (16·4%)	−6·7	18·7	0·70 (0·39 – 1·27)
35 – 44	125/1714 (7·3%)	169/1656 (10·2%)	−24·0	70·6	0·71 (0·52 – 0·97)
45 – 54	48/1521 (3·2%)	89/1548 (5·7%)	−20·9	33·4	0·53 (0·34 – 0·83)
55+	0/11 (0·0%)	0/17 (0·0%)			
**(d) BMI** (trend χ^2_1 = 1.0; 2p > 0.1; NS)					
<20	12/378 (3·2%)	25/399 (6·3%)	−7·9	8·6	0·40 (0·16 – 0·95)
20 – 24	82/1570 (5·2%)	123/1578 (7·8%)	−19·4	49·2	0·67 (0·47 – 0·97)
25 – 29	62/885 (7·0%)	88/841 (10·5%)	−17·0	34·7	0·61 (0·40 – 0·95)
30+	43/576 (7·5%)	57/568 (10·0%)	−6·7	23·1	0·75 (0·44 – 1·28)
**(e) Nodal status** (trend χ^2_1 = 11.1; 2p = 0.0009)					
N0	54/2132 (2·5%)	110/2099 (5·2%)	−29·1	40·4	0·49 (0·32 – 0·73)
N1−3	63/1014 (6·2%)	112/1049 (10·7%)	−24·8	42·5	0·56 (0·38 – 0·83)
N4+	90/379 (23·7%)	82/350 (23·4%)	0·9	40·1	
**(f) PR status** (χ^2_1 = 0.2; 2p > 0.1; NS)					
PR−	20/184 (10·9%)	24/175 (13·7%)	−2·9	9·7	
PR+	186/3325 (5·6%)	274/3308 (8·3%)	−47·9	112·7	0·65 (0·51 – 0·83)
**(g) Tumour diameter** (trend χ^2_1 = 0.0; 2p > 0.1; NS)					
1 – 20 mm (T1)	81/2345 (3·5%)	128/2331 (5·5%)	−24·6	51·2	0·62 (0·43 – 0·89)
21 – 50 mm (T2)	109/1022 (10·7%)	142/1011 (14·0%)	−16·8	59·6	0·75 (0·54 – 1·05)
> 50 mm (T3/T4)	13/107 (12·1%)	24/104 (23·1%)	−6·0	7·8	0·46 (0·18 – 1·17)
**(h) Tumour grade** (trend χ^2_1 = 1.1; 2p > 0.1; NS)					
Well−differentiated	13/623 (2·1%)	22/662 (3·3%)	−4·2	8·5	
Moderately	102/1994 (5·1%)	132/1988 (6·6%)	−13·6	56·4	0·79 (0·56 – 1·11)
Poorly	92/852 (10·8%)	146/806 (18·1%)	−30·2	56·3	0·58 (0·41 – 0·82)
**(i) Histological type** (χ^2_1 = 0.2; 2p > 0.1; NS)					
Ductal	176/2744 (6·4%)	255/2768 (9·2%)	−43·6	103·3	0·66 (0·51 – 0·84)
Lobular	15/379 (4·0%)	22/361 (6·1%)	−3·1	8·4	
Unknown	17/405 (4·2%)	27/373 (7·2%)	−5·5	10·4	
**(j) HER2 status** (χ^2_1 = 3.9; 2p = 0.05)					
HER2 positive	41/362 (11·3%)	40/361 (11·1%)	−1·4	18·7	
HER2 negative	136/2303 (5·9%)	235/2291 (10·3%)	−51·9	89·4	0·56 (0·43 – 0·73)
HER2 unknown	31/863 (3·6%)	29/850 (3·4%)	0·2	14·5	
**(k) Chemotherapy use** (χ^2_1 = 0.3; 2p > 0.1; NS)					
Absence	52/1932 (2·7%)	82/1921 (4·3%)	−16·4	32·9	0·61 (0·39 – 0·95)
Presence	156/1596 (9·8%)	222/1581 (14·0%)	−36·1	94·4	0·68 (0·52 – 0·89)
**(l) Bisphosphonate use*** (χ^2_1 = 0.4; 2p > 0.1; NS)					
Absence	195/3081 (6·3%)	286/3061 (9·3%)	−49·5	115·8	0·65 (0·51 – 0·83)
Presence	7/398 (1·8%)	12/393 (3·1%)	−3·3	4·6	
**Total**	**208/3528 (5·9%)**	**304/3502 (8·7%)**	**−52·5**	**123·3**	**0·653 (0·548 – 0·779) 2p < 0·00001**

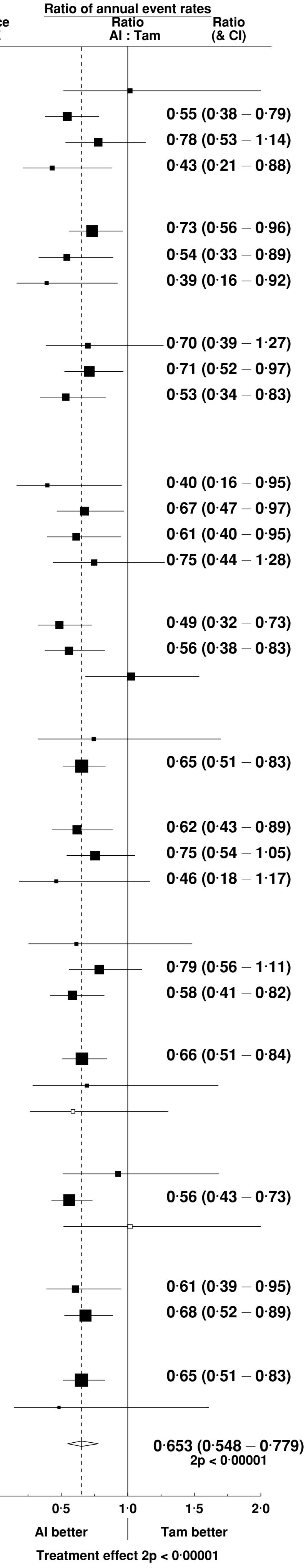

■ 99% or ◇ 95% confidence intervals

*Randomised bisphosphonate use

P8: Subgroup analyses of AI vs tamoxifen in premenopausal women; distant recurrence at any time

Category	Events/Women Allocated AI	Events/Women Allocated Tam	AI events Logrank O−E	AI events Variance of O−E	Ratio of annual event rates Ratio (& CI)
**(a) Follow up period, years (trend χ^2_1 = 0.1; 2p > 0.1; NS)**					
**0–1**	75/6851 (1·1%)	79/6816 (1·2%)	−3·0	37·2	0·92 (0·61 − 1·41)
**2–4**	115/9487 (1·2%)	154/9303 (1·7%)	−22·1	64·8	0·71 (0·52 − 0·98)
**5–9**	93/9196 (1·0%)	97/8991 (1·1%)	−5·2	46·1	0·89 (0·67 − 1·19)
**10+**	3/487 (0·6%)	0/475 (0·0%)	1·4	0·7	
**(b) Age at entry (trend χ^2_1 = 0.2; 2p > 0.1; NS)**					
**Age <35**	48/282 (17·0%)	52/281 (18·5%)	−4·7	23·2	0·82 (0·48 − 1·39)
**35 – 44**	166/1714 (9·7%)	184/1656 (11·1%)	−12·3	84·4	0·86 (0·65 − 1·14)
**45 – 54**	69/1521 (4·5%)	93/1548 (6·0%)	−11·9	39·5	0·74 (0·49 − 1·11)
**55+**	3/11 (27·3%)	1/17 (5·9%)	0·2	0·7	
**(c) BMI (trend χ^2_1 = 1.6; 2p > 0.1; NS)**					
**<20**	16/378 (4·2%)	31/399 (7·8%)	−8·1	10·3	0·46 (0·20 − 1·02)
**20 – 24**	109/1570 (6·9%)	129/1578 (8·2%)	−9·7	57·2	0·84 (0·60 − 1·19)
**25 – 29**	84/885 (9·5%)	93/841 (11·1%)	−8·8	41·3	0·81 (0·54 − 1·21)
**30+**	65/576 (11·3%)	67/568 (11·8%)	−2·6	30·8	0·92 (0·58 − 1·46)
**Unknown**	12/119 (10·1%)	10/116 (8·6%)	0·5	4·5	
**(d) Nodal status (trend χ^2_1 = 2.4; 2p > 0.1; NS)**					
**N0**	79/2132 (3·7%)	96/2099 (4·6%)	−10·8	43·0	0·78 (0·52 − 1·15)
**N1–3**	92/1014 (9·1%)	132/1049 (12·6%)	−21·1	54·9	0·68 (0·48 − 0·96)
**N4+**	114/379 (30·1%)	102/350 (29·1%)	2·5	50·6	1·05 (0·73 − 1·51)
**N unknown**	1/3 (33·3%)	0/4 (0·0%)	0·5	0·2	
**(e) PR status (χ^2_1 = 0.7; 2p > 0.1; NS)**					
**PR−**	29/184 (15·8%)	26/175 (14·9%)	0·6	12·4	
**PR?**	2/19 (10·5%)	4/19 (21·1%)	−0·4	1·2	
**PR+**	255/3325 (7·7%)	300/3308 (9·1%)	−26·6	135·6	0·82 (0·66 − 1·03)
**(f) Tumour diameter (trend χ^2_1 = 1.3; 2p > 0.1; NS)**					
**1 – 20 mm (T1)**	115/2345 (4·9%)	128/2331 (5·5%)	−7·7	59·7	0·88 (0·63 − 1·23)
**21 – 50 mm (T2)**	143/1022 (14·0%)	165/1011 (16·3%)	−13·4	73·7	0·83 (0·62 − 1·12)
**> 50 mm (T3/T4)**	18/107 (16·8%)	25/104 (24·0%)	−6·2	8·9	0·50 (0·21 − 1·19)
**Other / unknown**	10/54 (18·5%)	12/56 (21·4%)	−1·4	4·7	
**(g) Tumour grade (trend χ^2_1 = 0.1; 2p > 0.1; NS)**					
**Well−differentiated**	18/623 (2·9%)	27/662 (4·1%)	−4·0	10·6	0·69 (0·31 − 1·51)
**Moderately**	142/1994 (7·1%)	154/1988 (7·7%)	−5·6	72·2	0·92 (0·68 − 1·25)
**Poorly**	124/852 (14·6%)	143/806 (17·7%)	−15·6	63·8	0·78 (0·57 − 1·08)
**Grade unknown**	2/59 (3·4%)	6/46 (13·0%)	−0·7	0·8	
**(h) Histological type (χ^2_1 = 0.1; 2p > 0.1; NS)**					
**Ductal**	227/2744 (8·3%)	267/2768 (9·6%)	−25·7	119·0	0·81 (0·64 − 1·02)
**Lobular**	24/379 (6·3%)	30/361 (8·3%)	−4·1	12·4	0·72 (0·41 − 1·25)
**Unknown**	35/405 (8·6%)	33/373 (8·8%)	−1·4	15·1	
**(i) HER2 status (χ^2_1 = 8.7; 2p = 0.003)**					
**HER2+**	53/362 (14·6%)	36/361 (10·0%)	6·2	20·8	1·35 (0·77 − 2·37)
**HER2−**	166/2303 (7·2%)	237/2291 (10·3%)	−40·1	97·0	0·66 (0·51 − 0·86)
**HER2 unknown**	67/863 (7·8%)	57/850 (6·7%)	3·6	30·1	
**(j) Chemotherapy use (χ^2_1 = 1.1; 2p > 0.1; NS)**					
**Absence**	82/1932 (4·2%)	82/1921 (4·3%)	−1·8	40·1	0·96 (0·64 − 1·44)
**Presence**	204/1596 (12·8%)	248/1581 (15·7%)	−26·0	108·1	0·79 (0·61 − 1·01)
**■ Total**	**286/ 3528 (8·1%)**	**330/ 3502 (9·4%)**	**−28·9**	**148·8**	**0·823 (0·701 − 0·967)** 2p = 0·02

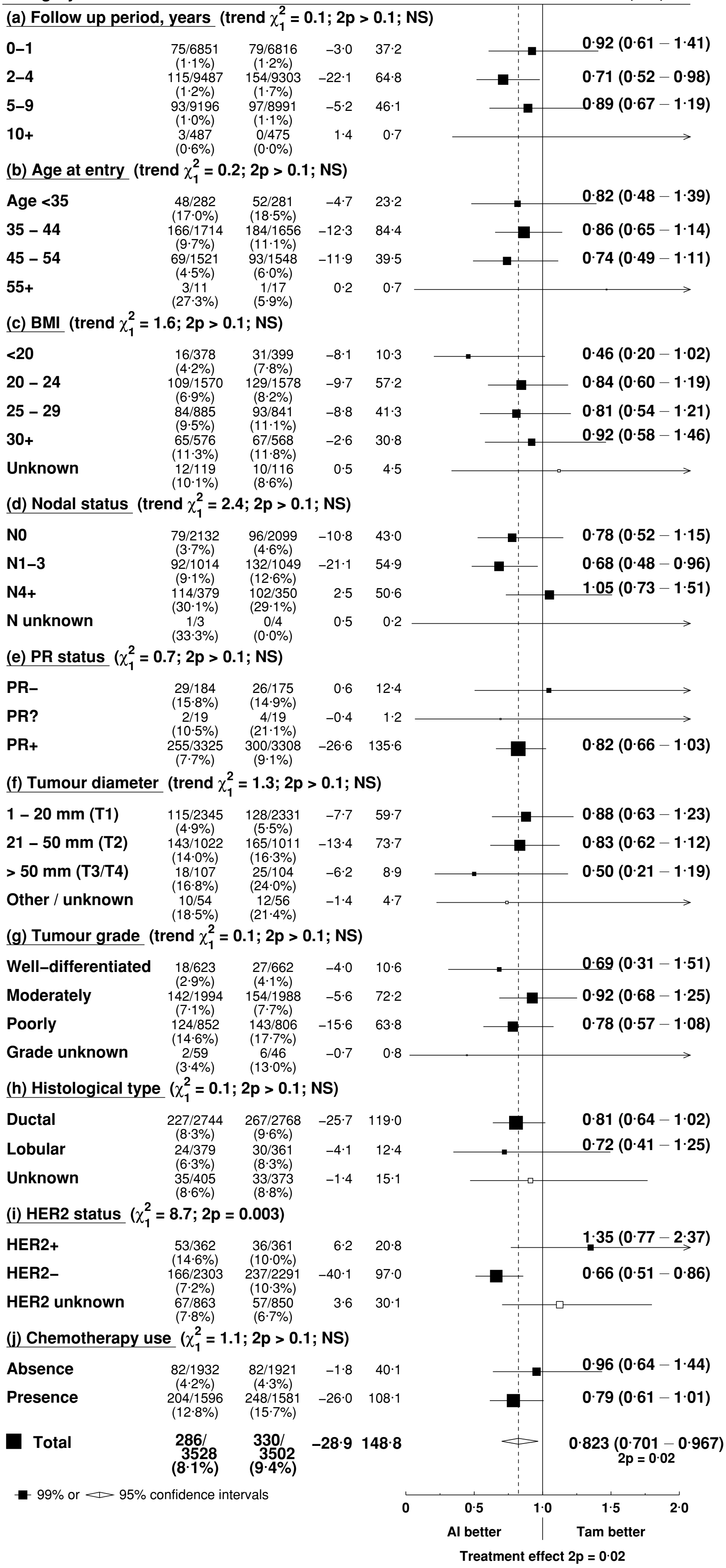

P9: Subgroup analyses of AI vs tamoxifen in premenopausal women; breast cancer mortality

Category	Events/Women: Allocated AI	Events/Women: Allocated Tam	AI events: Logrank O–E	AI events: Variance of O–E	Ratio (& CI)
**(a) Follow up period, years (trend χ^2_1 = 3.4; 2p = 0.07)**					
**0–1**	15/6900 (0·2%)	9/6876 (0·1%)	2·6	5·9	
**2–4**	85/9738 (0·9%)	69/9651 (0·7%)	7·1	37·5	1·21 (0·79 – 1·84)
**5–9**	81/9546 (0·8%)	98/9505 (1·0%)	–9·6	43·5	0·80 (0·54 – 1·19)
**10+**	4/513 (0·8%)	3/512 (0·6%)	0·6	1·7	
**(b) Age at entry (trend χ^2_1 = 0.1; 2p > 0.1; NS)**					
**Age <35**	34/282 (12·1%)	35/281 (12·5%)	–1·2	15·6	0·93 (0·48 – 1·78)
**35 – 44**	101/1714 (5·9%)	95/1656 (5·7%)	1·8	47·8	1·04 (0·72 – 1·51)
**45 – 54**	48/1521 (3·2%)	49/1548 (3·2%)	0·7	23·8	1·03 (0·61 – 1·75)
**55+**	2/11 (18·2%)	0/17 (0·0%)	0·4	0·2	
**(c) BMI (trend χ^2_1 = 1.1; 2p > 0.1; NS)**					
**<20**	11/378 (2·9%)	15/399 (3·8%)	–2·8	6·1	0·93 (0·48 – 1·78)
**20 – 24**	69/1570 (4·4%)	74/1578 (4·7%)	–1·7	34·6	0·95 (0·61 – 1·48)
**25 – 29**	54/885 (6·1%)	48/841 (5·7%)	0·9	23·9	1·04 (0·61 – 1·76)
**30+**	46/576 (8·0%)	39/568 (6·9%)	2·1	20·0	1·11 (0·62 – 1·98)
**Unknown**	5/119 (4·2%)	3/116 (2·6%)	–0·1	1·4	
**(d) Nodal status (trend χ^2_1 = 1.7; 2p > 0.1; NS)**					
**N0**	47/2132 (2·2%)	50/2099 (2·4%)	–2·0	24·0	0·92 (0·54 – 1·56)
**N1–3**	58/1014 (5·7%)	69/1049 (6·6%)	–5·5	31·1	0·84 (0·53 – 1·33)
**N4+**	79/379 (20·8%)	60/350 (17·1%)	7·7	33·3	1·26 (0·81 – 1·97)
**N unknown**	1/3 (33·3%)	0/4 (0·0%)	0·5	0·2	
**(e) PR status (χ^2_1 = 0.3; 2p > 0.1; NS)**					
**PR–**	19/184 (10·3%)	16/175 (9·1%)	1·7	8·1	
**PR?**	2/19 (10·5%)	1/19 (5·3%)	0·5	0·8	
**PR+**	164/3325 (4·9%)	162/3308 (4·9%)	0·1	79·9	1·00 (0·75 – 1·34)
**(f) Tumour diameter (trend χ^2_1 = 0.4; 2p > 0.1; NS)**					
**1 – 20 mm (T1)**	66/2345 (2·8%)	65/2331 (2·8%)	0·3	32·1	1·01 (0·64 – 1·59)
**21 – 50 mm (T2)**	102/1022 (10·0%)	91/1011 (9·0%)	5·5	46·5	1·31 (0·77 – 1·64)
**> 50 mm (T3/T4)**	10/107 (9·3%)	15/104 (14·4%)	–3·8	5·1	0·48 (0·15 – 1·49)
**Other / unknown**	7/54 (13·0%)	8/56 (14·3%)	–0·6	3·4	
**(g) Tumour grade (trend χ^2_1 = 0.5; 2p > 0.1; NS)**					
**Well–differentiated**	11/623 (1·8%)	8/662 (1·2%)	1·5	4·6	
**Moderately**	85/1994 (4·3%)	83/1988 (4·2%)	2·3	41·0	1·06 (0·71 – 1·58)
**Poorly**	88/852 (10·3%)	86/806 (10·7%)	–1·5	41·8	0·97 (0·65 – 1·44)
**Grade unknown**	1/59 (1·7%)	2/46 (4·3%)	0·2	0·5	
**(h) Histological type (χ^2_1 = 0.1; 2p > 0.1; NS)**					
**Ductal**	149/2744 (5·4%)	146/2768 (5·3%)	–1·2	71·5	0·98 (0·73 – 1·33)
**Lobular**	17/379 (4·5%)	18/361 (5·0%)	–0·8	8·3	
**Unknown**	19/405 (4·7%)	15/373 (4·0%)	0·9	7·7	
**(i) HER2 status (χ^2_1 = 3.3; 2p = 0.07)**					
**HER2+**	31/362 (8·6%)	19/361 (5·3%)	4·7	11·8	1·49 (0·70 – 3·15)
**HER2–**	108/2303 (4·7%)	130/2291 (5·7%)	–10·6	57·8	0·83 (0·59 – 1·17)
**HER2 unknown**	46/863 (5·3%)	30/850 (3·5%)	6·5	18·4	
**(j) Chemotherapy use (χ^2_1 = 1.7; 2p > 0.1; NS)**					
**Absence**	56/1932 (2·9%)	42/1921 (2·2%)	5·8	23·9	1·27 (0·75 – 2·16)
**Presence**	129/1596 (8·1%)	137/1581 (8·7%)	–4·5	64·5	0·93 (0·68 – 1·29)
**■ Total**	**185/ 3528 (5·2%)**	**179/ 3502 (5·1%)**	**0·7**	**88·6**	**1·008 (0·819 – 1·242)** 2p > 0·1; NS adverse

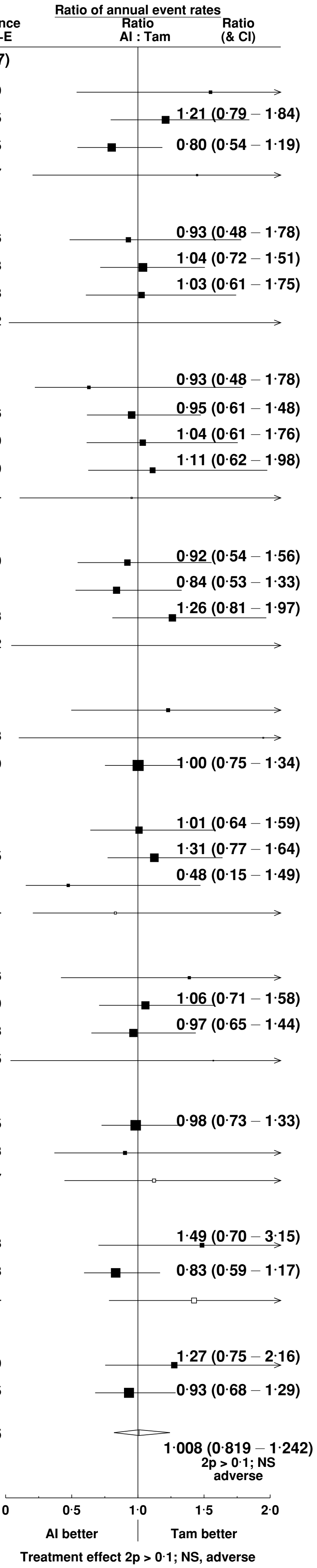

■ 99% or ◇ 95% confidence intervals

Treatment effect 2p > 0·1; NS, adverse

P10: Subgroup analyses of AI vs tamoxifen in premenopausal women; any recurrence by ER and PR

Category	Events/Women: Allocated AI	Events/Women: Allocated Tam	AI events: Logrank O−E	AI events: Variance of O−E	Ratio (& CI)
**ER+ PR+**	365/3325 (11·0%)	444/3308 (13·4%)	−45·4	197·9	**0·80 (0·66 – 0·95)**
**ER+ PR−**	36/184 (19·6%)	36/175 (20·6%)	−1·2	16·3	
**ER+ PR?**	3/19 (15·8%)	4/19 (21·1%)	0·1	1·5	
**ER− PR+**	11/43 (25·6%)	7/48 (14·6%)	2·2	3·8	
**ER− PR−**	5/19 (26·3%)	9/30 (30·0%)	0·1	2·5	
**ER? PR any**	0/28 (0·0%)	0/32 (0·0%)			
**Total**	**420/ 3618 (11·6%)**	**500/ 3612 (13·8%)**	**−44·2**	**222·1**	**0·819 (0·718 – 0·935)** 2p = 0·003

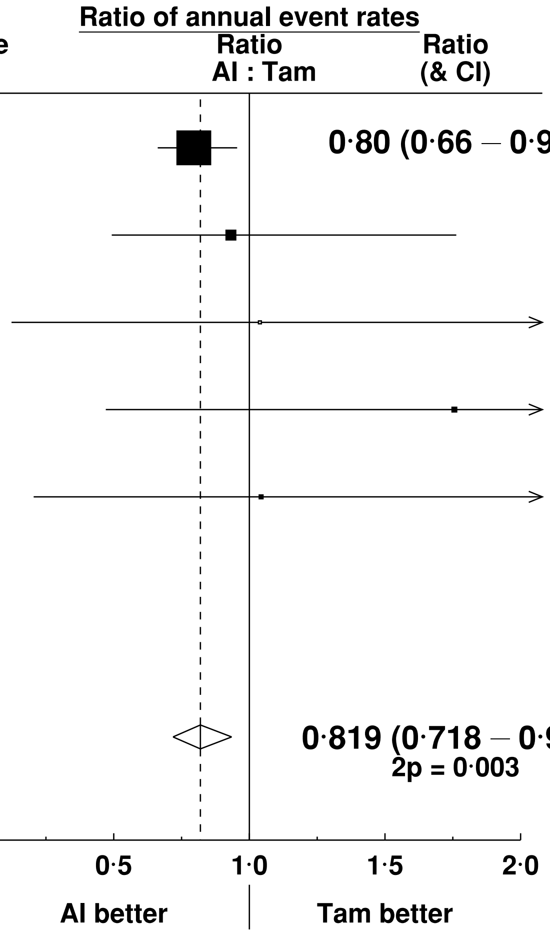

99% or 95% confidence intervals

Heterogeneity between 4 categories: χ^2_3 = 2·8; p > 0·1; NS

Treatment effect 2p = 0·003

P11: 10 year cumulative risk recurrence by PR status with smoothing from year 5

ER+, PR−

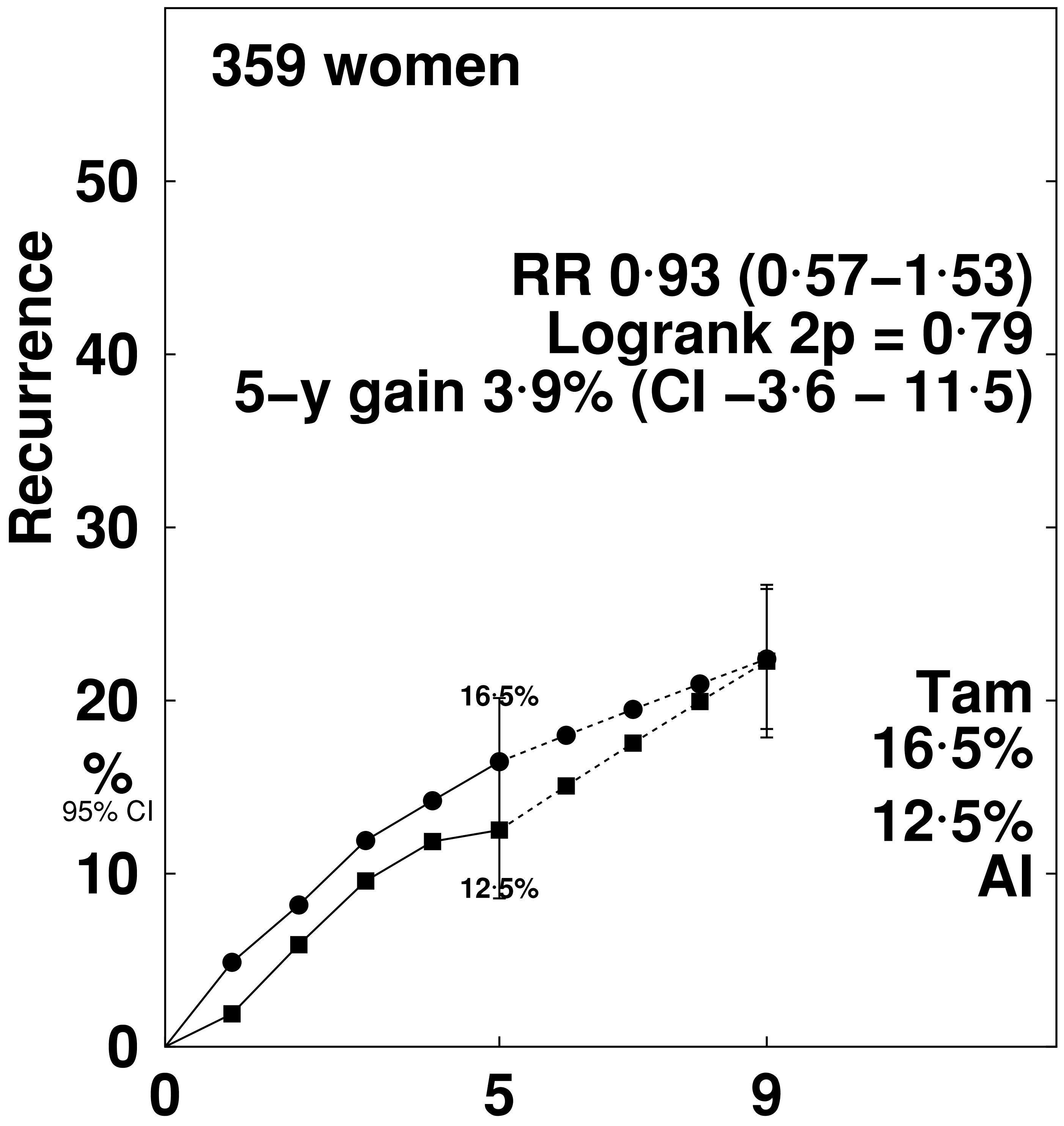

Recurrence rates (% / year) and logrank analyses

Allocation	Years 0 – 4	Year 5+
AI	2·80 (23 / 821)	2·91 (13 / 446)
Tam	3·57 (28 / 785)	1·86 (8 / 431)
Rate ratio, from	0·75 CI 0·42 – 1·34	1·65 CI 0·64 – 4·20
(O–E) / V	–3·3 / 11·5	2·2 / 4·4

ER+, PR+

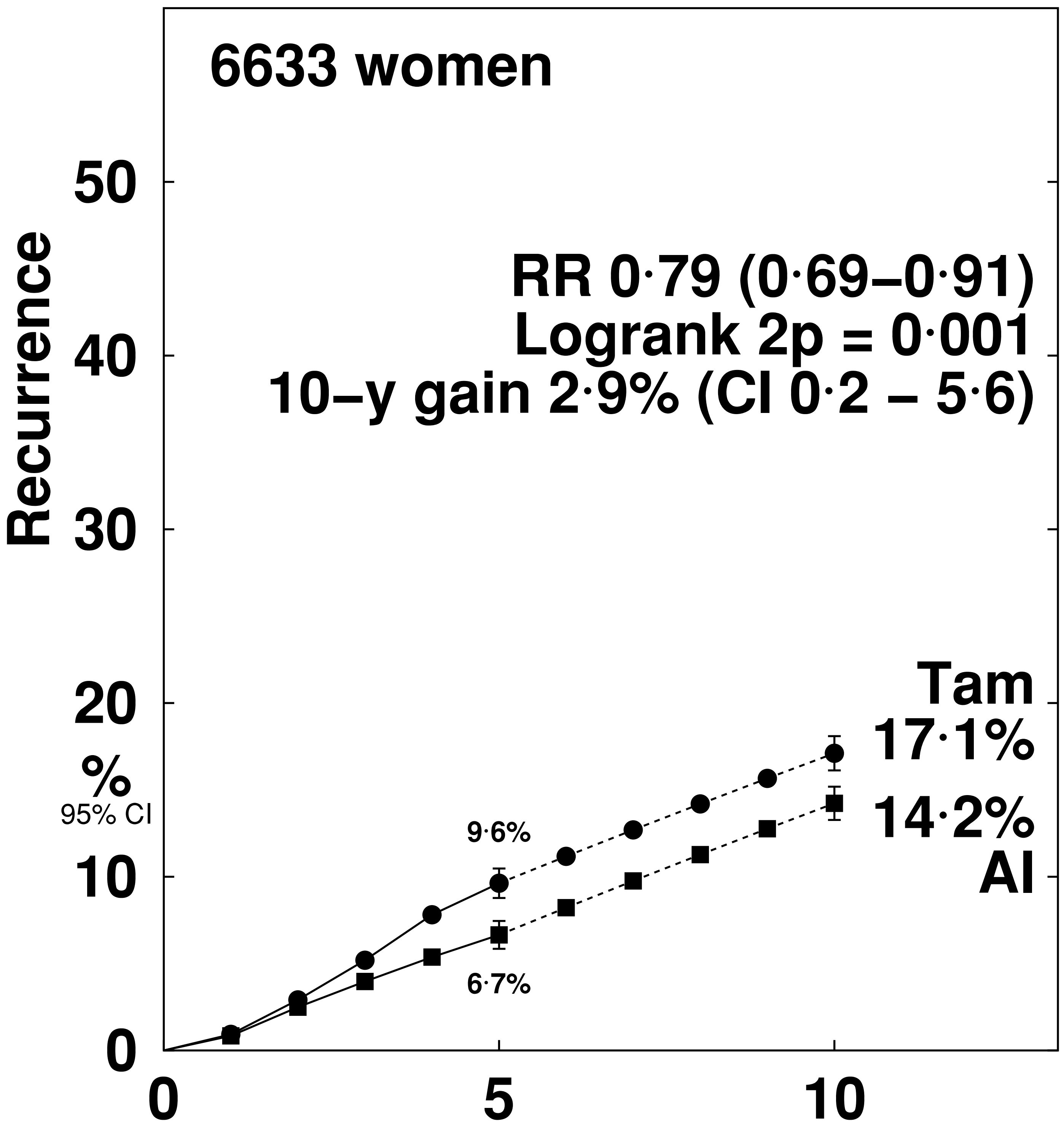

Recurrence rates (% / year) and logrank analyses

Allocation	Years 0 – 4	Years 5 – 9	Year 10+
AI	1·38 (212 / 15368)	1·70 (145 / 8524)	1·74 (8 / 461)
Tam	2·00 (302 / 15096)	1·72 (141 / 8197)	0·23 (1 / 438)
Rate ratio, from	0·69 CI 0·57 – 0·82	0·98 CI 0·77 – 1·24	4·44 CI 1·19 – 16·59
(O–E) / V	–47·2 / 124·9	–1·5 / 69·0	3·3 / 2·2

P12: 10 year cumulative risk recurrence by nodal status smoothed from year 5

ER+, N0

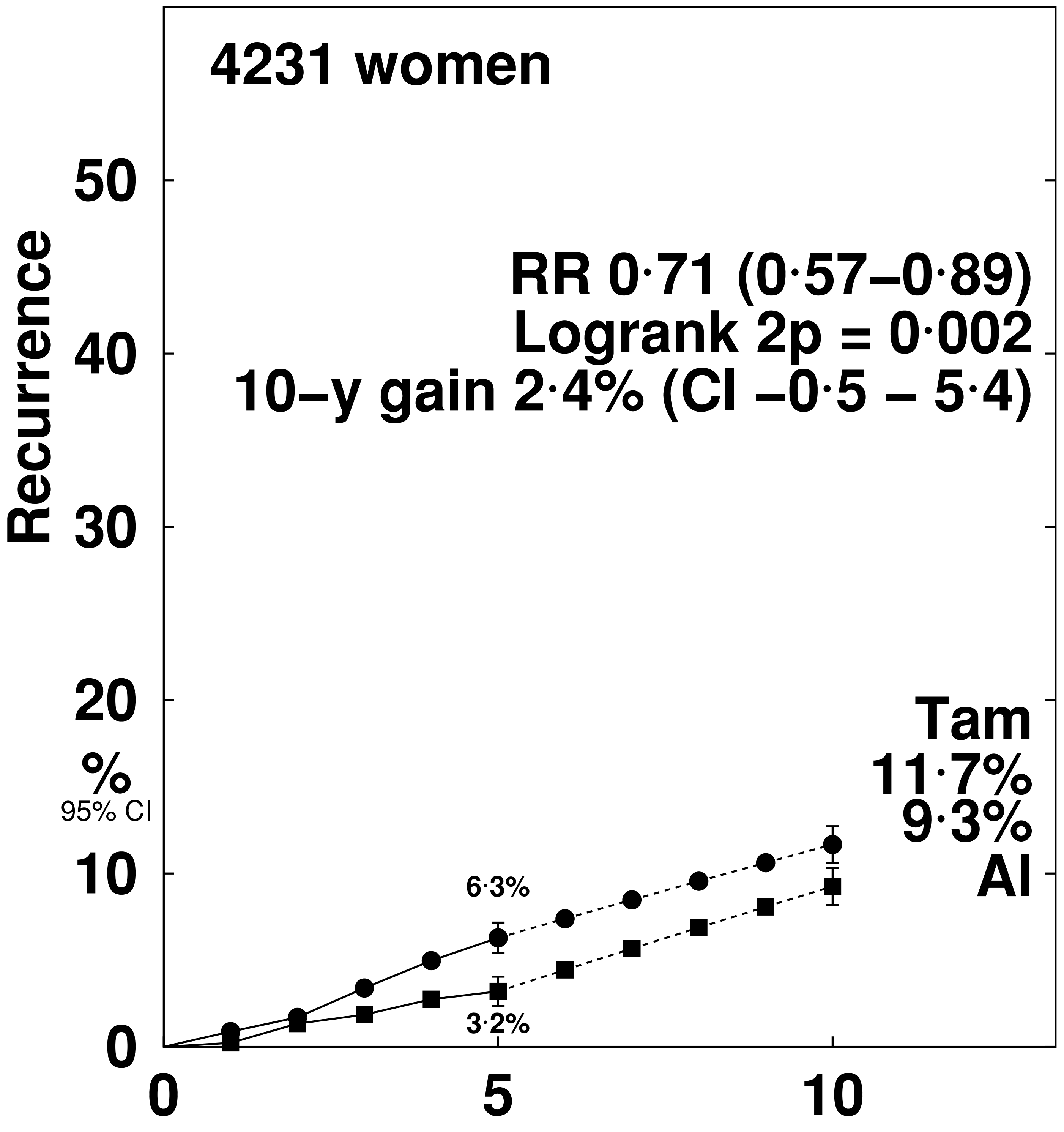

Recurrence rates (% / year) and logrank analyses

Allocation	Years 0 – 4	Years 5 – 9	Year 10+
AI	0·66 (66 / 9994)	1·33 (74 / 5575)	1·48 (4 / 270)
Tam	1·28 (125 / 9741)	1·14 (61 / 5328)	0·71 (2 / 280)
Rate ratio, from	0·52 CI 0·39 – 0·69	1·09 CI 0·78 – 1·54	1·78 CI 0·35 – 8·98
(O–E) / V	–31·2 / 47·0	2·9 / 32·8	0·8 / 1·5

ER+, N1–3

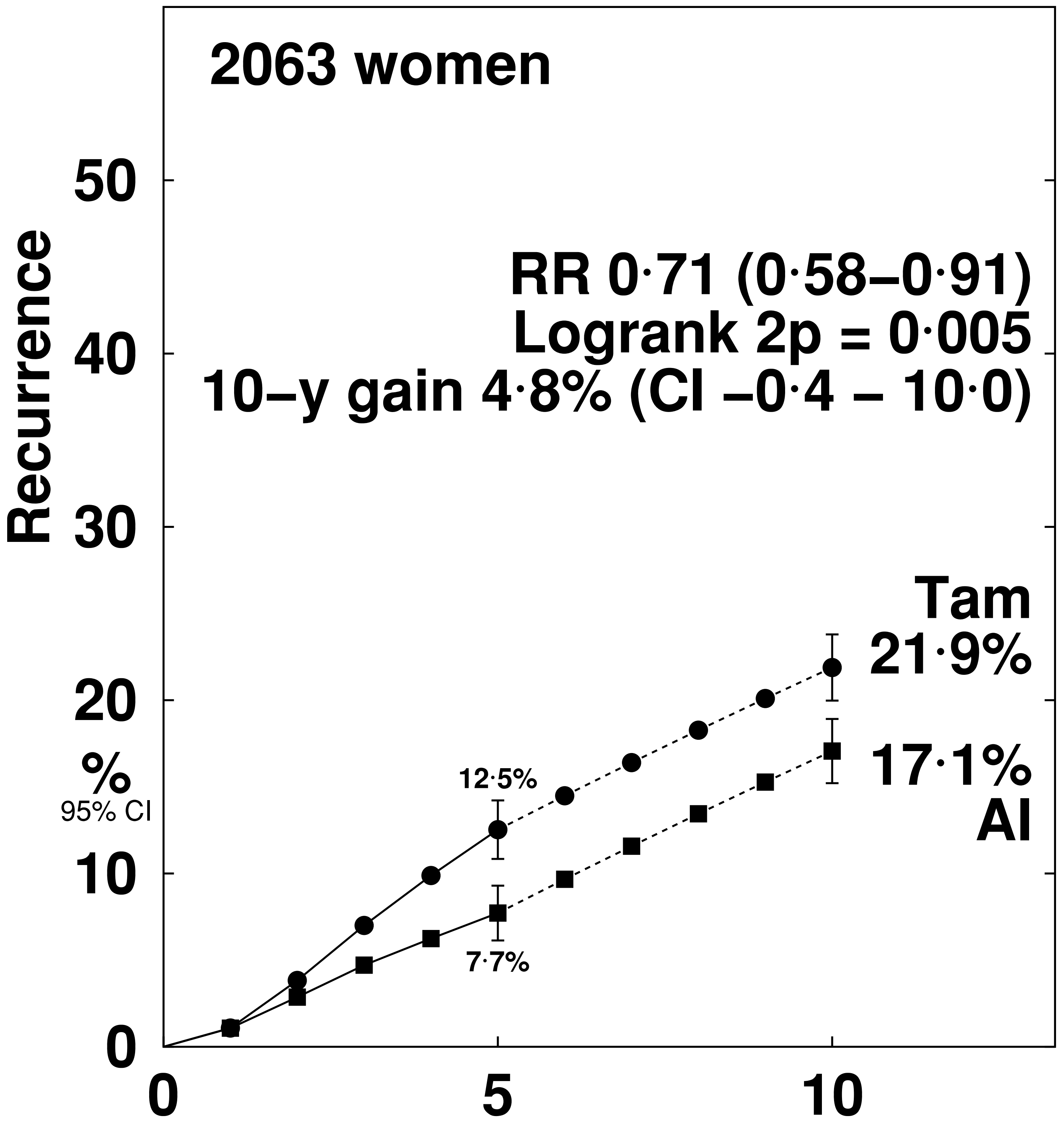

Recurrence rates (% / year) and logrank analyses

Allocation	Years 0 – 4	Years 5 – 9	Year 10+
AI	1·62 (76 / 4697)	2·14 (57 / 2661)	1·21 (2 / 165)
Tam	2·62 (124 / 4729)	2·26 (58 / 2567)	0·0(0/130)
Rate ratio, from	0·61 CI 0·46 – 0·81	0·94 CI 0·65 – 1·37	6·69 CI 0·42 – 107·56
(O–E) / V	–24·2 / 48·6	–1·6 / 27·8	0·9 / 0·5

ER+, N4+

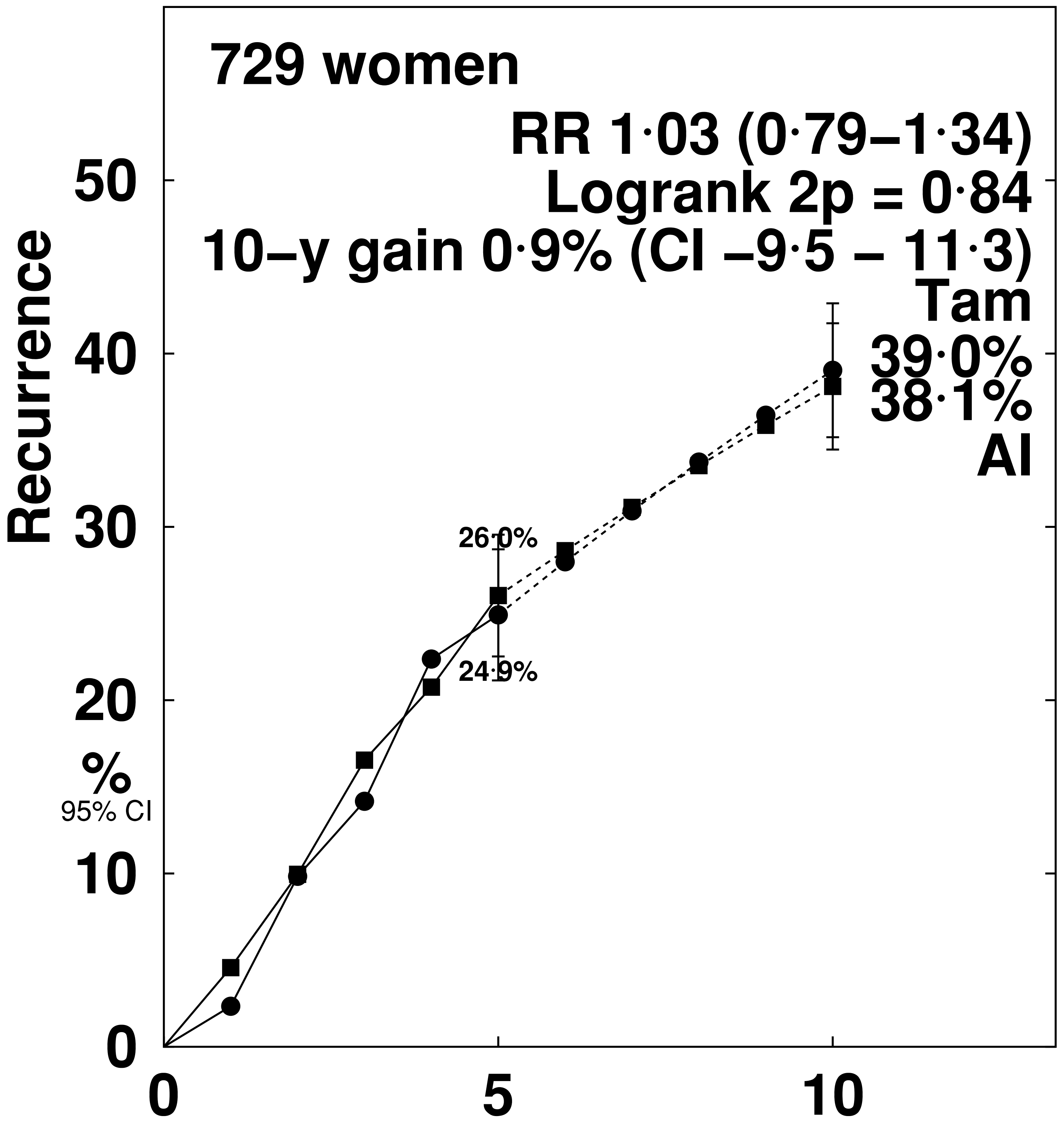

Recurrence rates (% / year) and logrank analyses

Allocation	Years 0 – 4	Years 5 – 9	Year 10+
AI	5·98 (94 / 1572)	3·55 (27 / 761)	7·50 (3 / 40)
Tam	5·62 (83 / 1477)	4·19 (31 / 740)	0·0(0/47)
Rate ratio, from	1·06 CI 0·78 – 1·44	0·85 CI 0·50 – 1·46	7·62 CI 0·70 – 83·24
(O–E) / V	2·3 / 41·2	–2·1 / 13·5	1·4 / 0·7

P13: 10 year cumulative risk recurrence by tumour grade smoothed from year 5

ER+, Well–differentiated

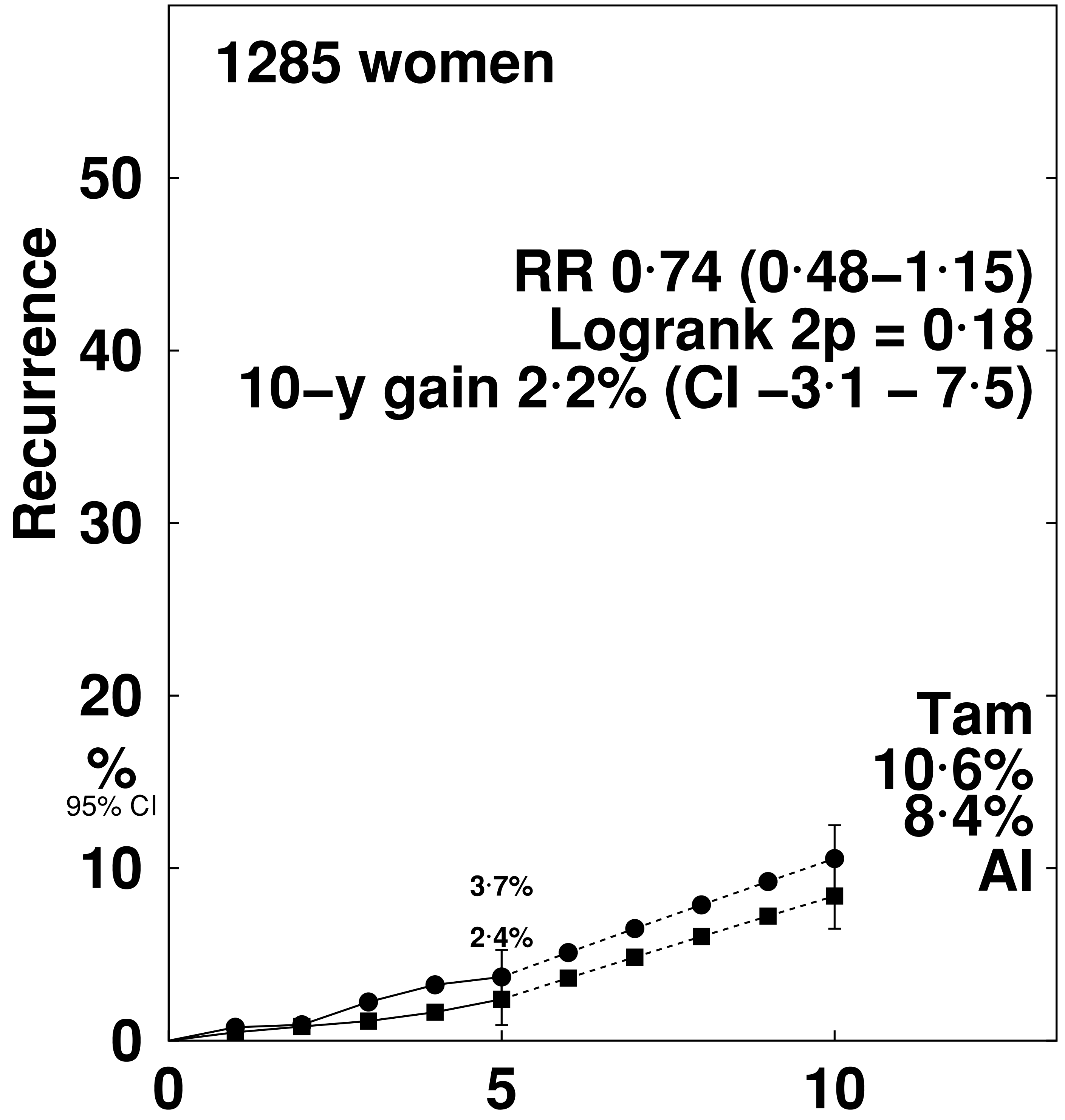

Recurrence rates (% / year) and logrank analyses

Allocation	Years 0 – 4	Years 5 – 9	Year 10+
AI	0·48 (14 / 2935)	1·30 (22 / 1695)	0·0(0/116)
Tam	0·75 (23 / 3070)	1·44 (26 / 1804)	0·77 (1 / 130)
Rate ratio, from	0·65 CI 0·34 – 1·24	0·86 CI 0·47 – 1·56	0·21 CI 0·00 – 13·02
(O–E) / V	–3·9 / 9·0	–1·6 / 10·6	–0·4 / 0·2

ER+, Moderately

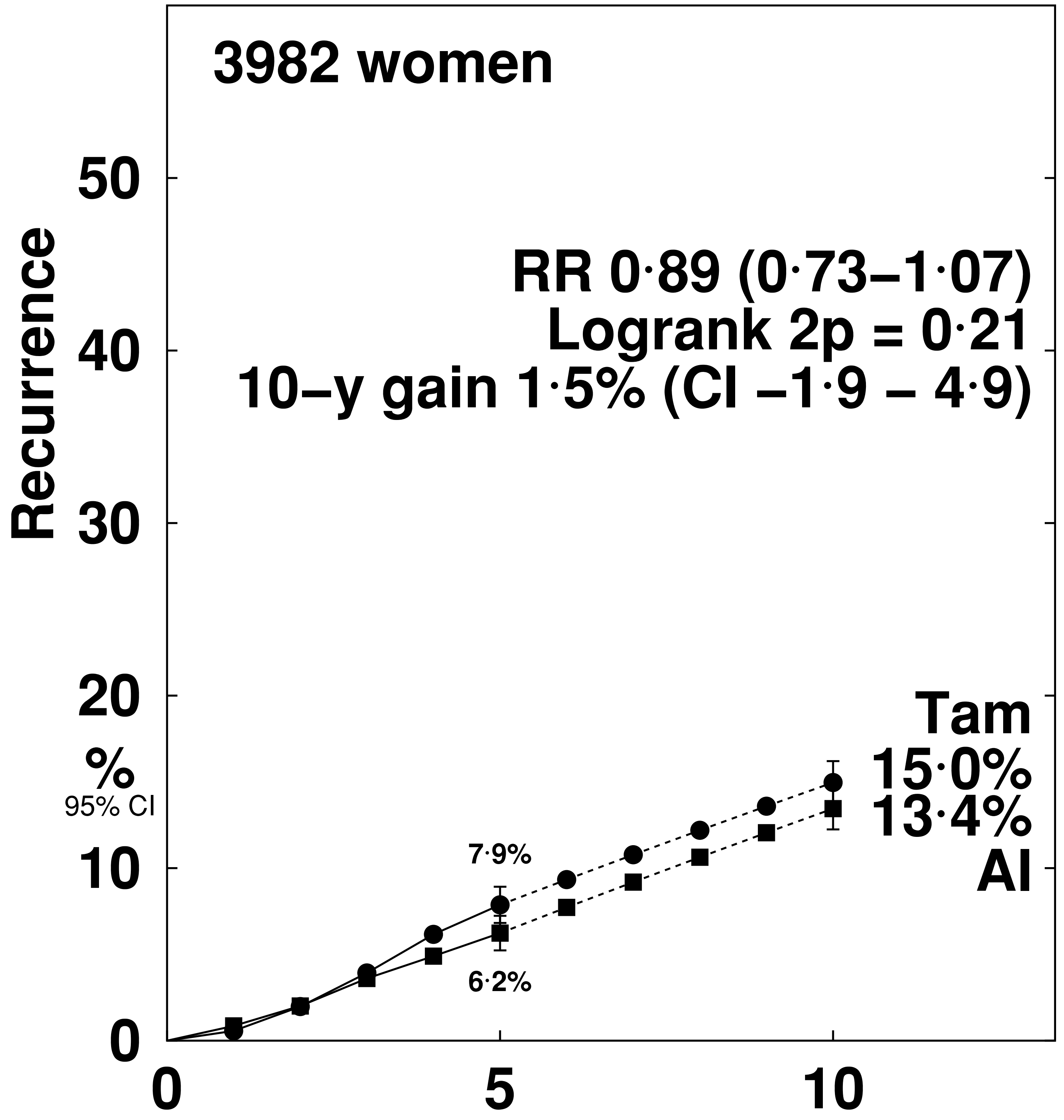

Recurrence rates (% / year) and logrank analyses

Allocation	Years 0 – 4	Years 5 – 9	Year 10+
AI	1·26 (117 / 9257)	1·59 (81 / 5097)	1·95 (5 / 256)
Tam	1·63 (150 / 9183)	1·61 (80 / 4955)	0·0(0/241)
Rate ratio, from	0·79 CI 0·62 – 1·01	1·00 CI 0·73 – 1·37	7·04 CI 1·18 – 41·87
(O–E) / V	–15·1 / 64·5	–0·0 / 39·1	2·4 / 1·2

ER+, Poorly

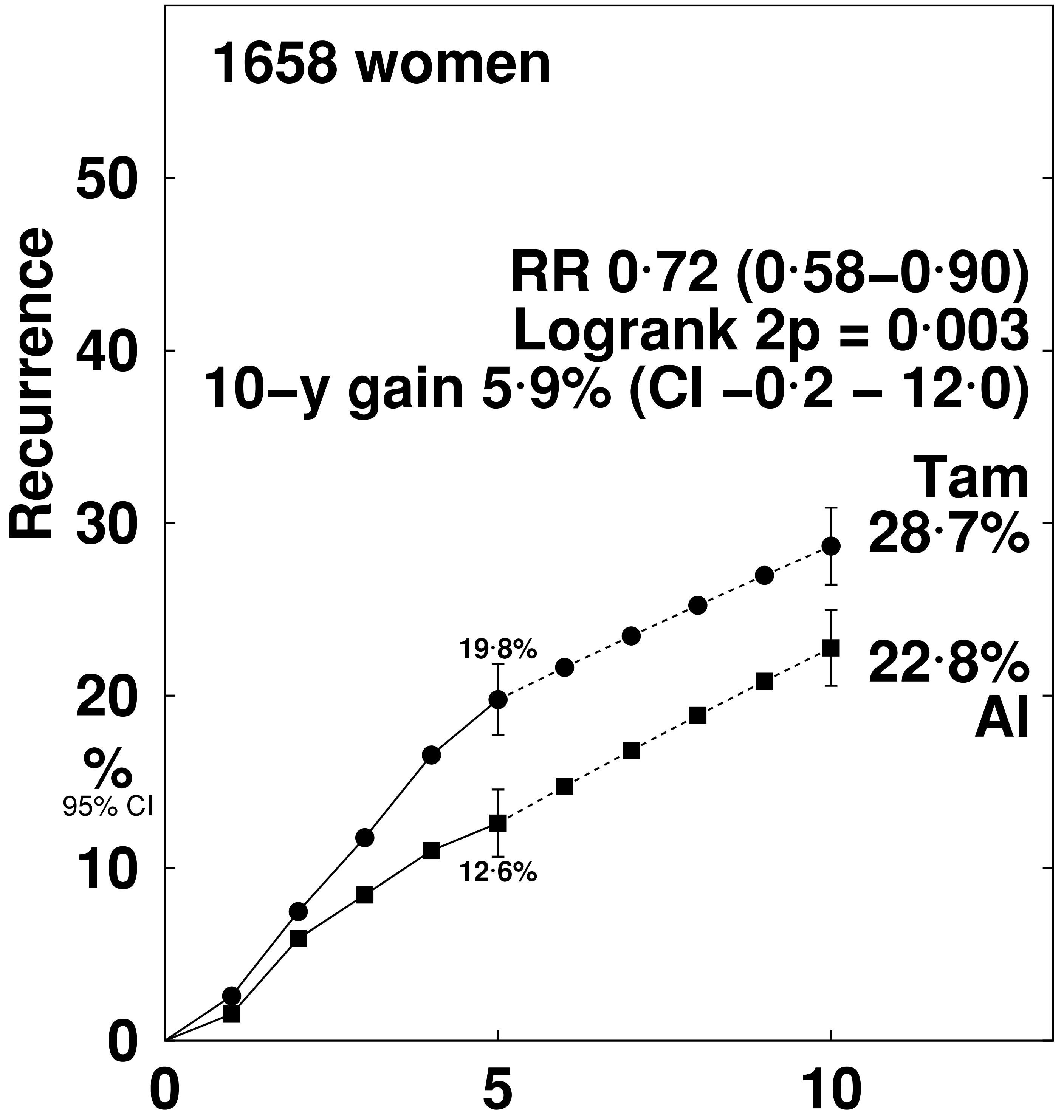

Recurrence rates (% / year) and logrank analyses

Allocation	Years 0 – 4	Years 5 – 9	Year 10+
AI	2·74 (105 / 3828)	2·53 (53 / 2091)	4·04 (4 / 99)
Tam	4·41 (155 / 3516)	2·27 (41 / 1807)	1·16 (1 / 86)
Rate ratio, from	0·62 CI 0·48 – 0·80	1·05 CI 0·69 – 1·60	2·46 CI 0·40 – 15·11
(O–E) / V	–29·5 / 61·5	1·1 / 21·9	1·0 / 1·2

P14: Any recurrence in AI versus tamoxifen in premenopausal women: (A) in the presence and absence absence chemotherapy, (B) in the presence and absence of bisphosphonate

(A) Chemotherapy

Year code and study name	Treatment comparison	Events/Women: Allocated AI	Events/Women: Allocated Tam	AI events: Logrank O–E	AI events: Variance of O–E
**(a) AI vs tam in the absence of chemotherapy**					
99B1+2 Austrian BCSG XII	Gos;(Ana vs Tam)±Zol 3yr	102/806	88/791	4·7	45·8
03<2 TEXT / IBCSG 25-02	Trip;(Exe vs Tam) 5yr	32/528	45/529	−7·5	19·0
03E2 SOFT / IBCSG 24-02	Trip;(Exe vs Tam) 5yr	24/469	34/469	−5·5	14·2
04]5 IT Naples HOBOE	Trip;(Let vs Tam) 5yr	3/129	9/132	−3·0	3·0
**(a) subtotal**		**161/ 1932 (8·3%)**	**176/ 1921 (9·2%)**	**−11·4**	**82·0**
**(b) AI vs tam in the presence of chemotherapy**					
99B5+6 Austrian BCSG XII	Gos;(Ana vs Tam)±Zol 3yr	12/49	15/48	−1·0	5·9
03<1 TEXT / IBCSG 25-02	Trip;(Exe vs Tam) 5yr	105/796	150/782	−28·4	61·4
03E1 SOFT / IBCSG 24-02	Trip;(Exe vs Tam) 5yr	91/530	105/530	−5·7	46·9
04]1 IT Naples HOBOE	Trip;(Let vs Tam) 5yr	35/221	38/221	−3·8	17·5
**(b) subtotal**		**243/ 1596 (15·2%)**	**308/ 1581 (19·5%)**	**−38·9**	**131·7**
**Total (a + b)**		**404/ 3528 (11·5%)**	**484/ 3502 (13·8%)**	**−50·3**	**213·7**

Heterogeneity between 4 trials (a): $\chi^2_3 = 7{\cdot}1$; p = 0·07

Heterogeneity between 4 trials (b): $\chi^2_3 = 3{\cdot}3$; p > 0·1; NS

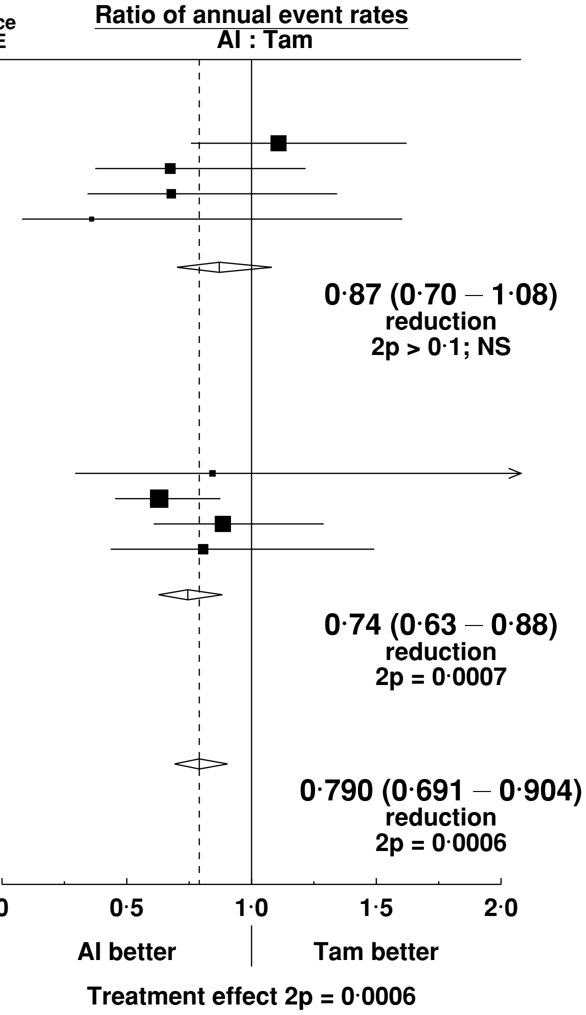

99% or 95% confidence intervals

Difference between treatment effects in 2 subtotals: $\chi^2_1 = 1{\cdot}2$; 2p > 0·1; NS

Heterogeneity within subtotals: $\chi^2_6 = 10{\cdot}4$; p > 0·1; NS

Heterogeneity between 8 trials: $\chi^2_7 = 11{\cdot}7$; p > 0·1; NS

(B) Randomised bisphosphonate use

Year code and study name	Treatment comparison	Events/Women: Allocated AI	Events/Women: Allocated Tam	AI events: Logrank O–E	AI events: Variance of O–E
**(a) Bisphosphonates given**					
99B1256 Austrian BCSG XII	Gos;(Ana vs Tam)+Zol 3yr	51/424	46/416	−0·6	22·0
**(a) subtotal**		**51/ 424 (12·0%)**	**46/ 416 (11·1%)**	**−0·6**	**22·0**
**(b) No systemic bisphosphonate use**					
99B1256 Austrian BCSG XII	Gos;(Ana vs Tam) 3yr	63/431	57/423	3·6	28·1
03< TEXT / IBCSG 25-02	Trip;(Exe vs Tam) 5yr	137/1324	195/1311	−35·9	80·4
03E SOFT / IBCSG 24-02	Trip;(Exe vs Tam) 5yr	115/999	139/999	−11·2	61·1
04]1+5 IT Naples HOBOE	Trip;(Let vs Tam) 5yr	38/350	47/353	−6·8	20·5
**(b) subtotal**		**353/ 3104 (11·4%)**	**438/ 3086 (14·2%)**	**−50·3**	**190·1**
**Total (a + b)**		**404/ 3528 (11·5%)**	**484/ 3502 (13·8%)**	**−51·0**	**212·1**

Heterogeneity between 4 trials (b): $\chi^2_3 = 7{\cdot}5$; p = 0·06

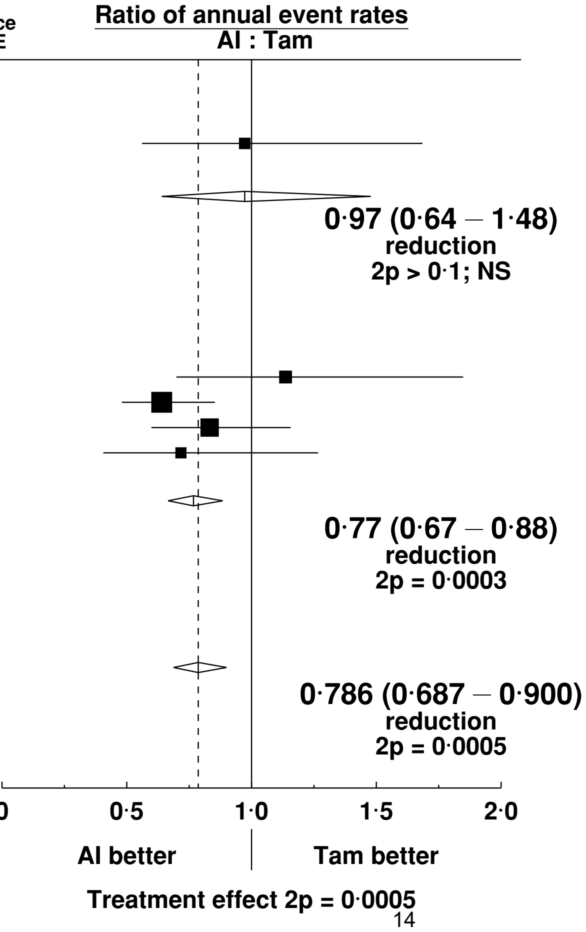

99% or 95% confidence intervals

Difference between treatment effects in 2 subtotals: $\chi^2_1 = 1{\cdot}1$; 2p > 0·1; NS

Heterogeneity within subtotals: $\chi^2_3 = 7{\cdot}5$; p = 0·06

Heterogeneity between 5 trials: $\chi^2_4 = 8{\cdot}6$; p = 0·07

P15: Any recurrence in AI versus tamoxifen in premenopausal women: in the presence and absence of chemotherapy (excluding ABCSG XII)

Year code and study name	Treatment comparison	Events/Women: Allocated AI	Events/Women: Allocated Tam	AI events: Logrank O–E	AI events: Variance of O–E
**(a) AI vs tam in the absence of chemotherapy**					
03<2 TEXT / IBCSG 25–02	Trip;(Exe vs Tam) 5yr	32/528	45/529	–7·5	19·0
03E2 SOFT / IBCSG 24–02	Trip;(Exe vs Tam) 5yr	24/469	34/469	–5·5	14·2
04]5 IT Naples HOBOE	Trip;(Let vs Tam) 5yr	3/129	9/132	–3·0	3·0
**(a) subtotal**		**59/ 1126 (5·2%)**	**88/ 1130 (7·8%)**	**–16·1**	**36·2**
**Heterogeneity between 3 trials: χ^2_2 = 1·1; p > 0·1; NS**					
**(b) AI vs tam in the presence of chemotherapy**					
03<1 TEXT / IBCSG 25–02	Trip;(Exe vs Tam) 5yr	105/796	150/782	–28·4	61·4
03E1 SOFT / IBCSG 24–02	Trip;(Exe vs Tam) 5yr	91/530	105/530	–5·7	46·9
04]1 IT Naples HOBOE	Trip;(Let vs Tam) 5yr	35/221	38/221	–3·8	17·5
**(b) subtotal**		**231/ 1547 (14·9%)**	**293/ 1533 (19·1%)**	**–37·9**	**125·8**
**Heterogeneity between 3 trials: χ^2_2 = 3·2; p > 0·1; NS**					
**Total (a + b)**		**290/ 2673 (10·8%)**	**381/ 2663 (14·3%)**	**–53·9**	**162·0**

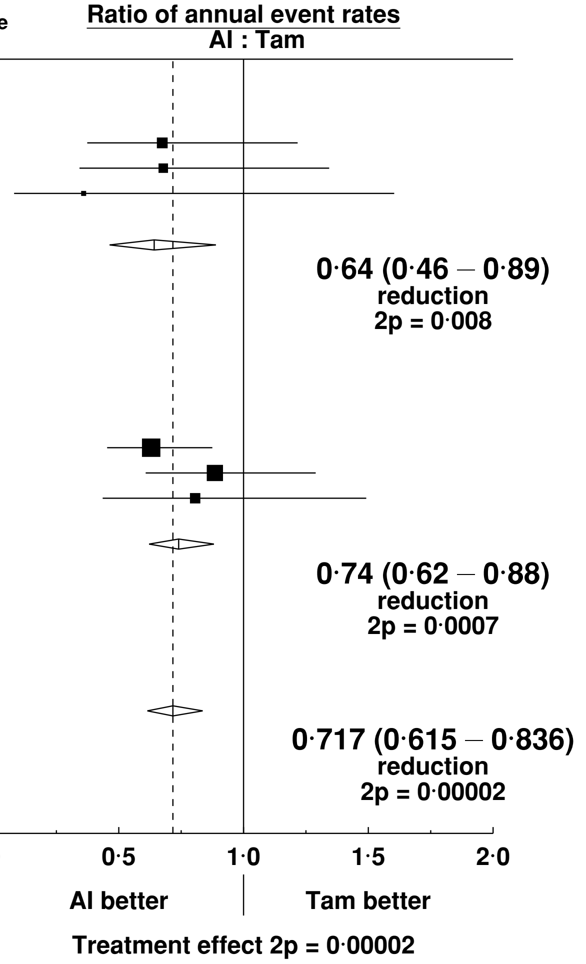

99% or 95% confidence intervals

Difference between treatment effects in 2 subtotals: χ^2_1 = 0·6; 2p > 0·1; NS

Heterogeneity within subtotals: χ^2_4 = 4·3; p > 0·1; NS

Heterogeneity between 6 trials: χ^2_5 = 4·9; p > 0·1; NS

P16: Mortality by cause, incidence of second cancers and bone fracture incidence

Cause of death	AI	Tam	(O-E)	Variance	RR	95% CI	p
**Cancers other than breast**	**22**	**10**	**6.6**	**7.4**	**2.44**	**(1.19 – 5.01)**	**0.02**
Haematological	3	1	1.0	1.0	2.7	(0.38 – 19.2)	0.32
Lung	6	4	1.0	2.5	1.49	(0.43 – 5.15)	0.53
Ovarian	4	1	1.5	1.3	3.17	(0.57 – 17.7)	0.19
Pancreatic	4	1	1.5	1.3	3.17	(0.57 – 17.7)	0.19
Other non-breast*	5	3	1.6	1.3	3.42	(0.61 – 19.1)	0.16
**Vascular disease**	**2**	**1**	**0.4**	**0.7**	**1.77**	**(0.17 – 18.4)**	**0.63**
**Other specified disease****	**3**	**9**	**-3.0**	**3.1**	**0.38**	**(0.13 – 1.16)**	**0.09**
**Unknown**	**3**	**4**	**-0.6**	**1.7**	**0.70**	**(0.16 – 3.16)**	**0.65**
**Total**	**30**	**24**	**3.4**	**12.9**	**1.30**	**(0.75 – 2.25)**	**0.34**

*Other non-breast cancer deaths in AI group: colorectal, gastric, head & neck, skin (2). In Tam group: brain, primary liver, uterine.

**Other specified disease in AI group: hepatic disease, other specified not breast cancer (2). In Tam group: diabetes, mixed drug intoxication, suicide (2), accident, infectious/parasitic, other specified not breast cancer (3).

SECOND PRIMARY CANCER INCIDENCE in trials of Aromatase inhibitor versus tamoxifen, ER+

Year code and study name	Treatment comparison	Events/Women Allocated AI	Events/Women Allocated Tam	AI events Logrank O–E	AI events Variance of O–E
**AI vs tamoxifen (premenopausal women with ovarian suppression)**					
99B1+2 Austrian BCSG XII	Gos;(Ana vs Tam)±Zol 3yr	62/855	44/839	7·5	25·8
03< TEXT / IBCSG 25–02	Trip;(Exe vs Tam) 5yr	31/1324	25/1311	3·2	13·7
03E SOFT / IBCSG 24–02	Trip;(Exe vs Tam) 5yr	20/999	21/999	–0·2	10·0
04]1+5 IT Naples HOBOE	Trip;(Let vs Tam) 5yr	11/350	21/353	–5·9	7·8
**■ Total**		**124/ 3528 (3·5%)**	**111/ 3502 (3·2%)**	**4·6**	**57·4**

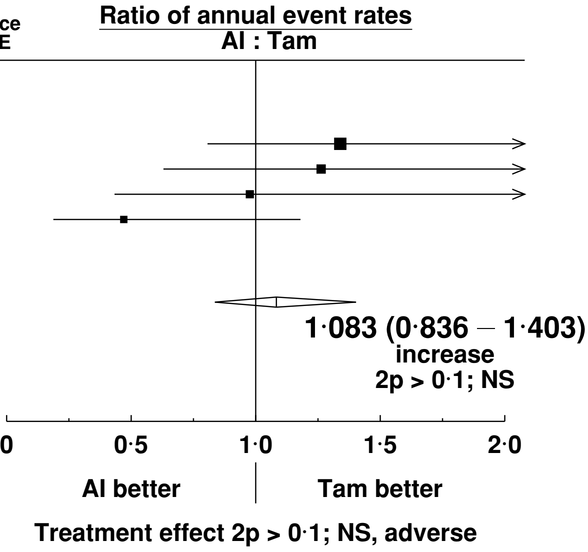

■ 99% or ◇ 95% confidence intervals

Heterogeneity between 4 trials: $\chi^2_3 = 7{\cdot}1$; p = 0·07

BONE FRACTURE INCIDENCE in trials of Aromatase inhibitor versus tamoxifen, ER+

Year code and study name	Treatment comparison	Events/Women Allocated AI	Events/Women Allocated Tam	AI events Logrank O–E	AI events Variance of O–E
**AI vs tamoxifen (premenopausal women with ovarian suppression)**					
99B1256 Austrian BCSG XII	Gos;(Ana vs Tam)±Zol 3yr	16/855	12/839	1·6	6·9
03< TEXT / IBCSG 25–02	Trip;(Exe vs Tam) 5yr	133/1324	99/1311	16·4	57·1
03E SOFT / IBCSG 24–02	Trip;(Exe vs Tam) 5yr	78/999	69/999	5·8	36·0
04]1+5 IT Naples HOBOE	Trip;(Let vs Tam) 5yr	0/350	0/353		
**■ Total**		**227/ 3528 (6·4%)**	**180/ 3502 (5·1%)**	**23·8**	**100·0**

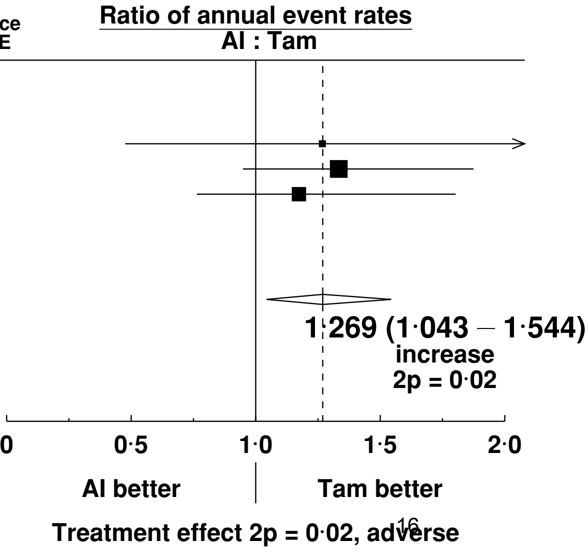

■ 99% or ◇ 95% confidence intervals

Heterogeneity between 3 trials: $\chi^2_2 = 0{\cdot}4$; p > 0·1; NS

P17: Death without recurrence by tumour size and nodal status

Category	Events/Women: Allocated AI	Events/Women: Allocated Tam	AI events: Logrank O−E	AI events: Variance of O−E	Ratio of annual event rates: Ratio (& CI)
**(a) Tumour size** (trend χ^2_1 = 8.5; 2p = 0.003)					
**1 – 20 mm (T1)**	23/2345 (1·0%)	9/2331 (0·4%)	6·8	7·7	
**21 – 50 mm (T2)**	6/1022 (0·6%)	14/1011 (1·4%)	−3·9	4·7	
**> 50 mm (T3/T4)**	0/107 (0·0%)	0/104 (0·0%)			
**Other / unknown**	1/54 (1·9%)	1/56 (1·8%)	−0·2	0·5	
**(b) Nodal status** (trend χ^2_1 = 1.8; 2p > 0.1; NS)					
**N0**	15/2132 (0·7%)	16/2099 (0·8%)	0·1	7·2	
**N1–3**	10/1014 (1·0%)	7/1049 (0·7%)	1·4	4·2	
**N4+**	5/379 (1·3%)	1/350 (0·3%)	1·9	1·5	
**N unknown**	0/3 (0·0%)	0/4 (0·0%)			
■ **Total**	**60/ 7056 (0·9%)**	**48/ 7004 (0·7%)**	**6·1**	**25·8**	**1·266 (0·861 – 1·863)** 2p > 0·1; NS adverse

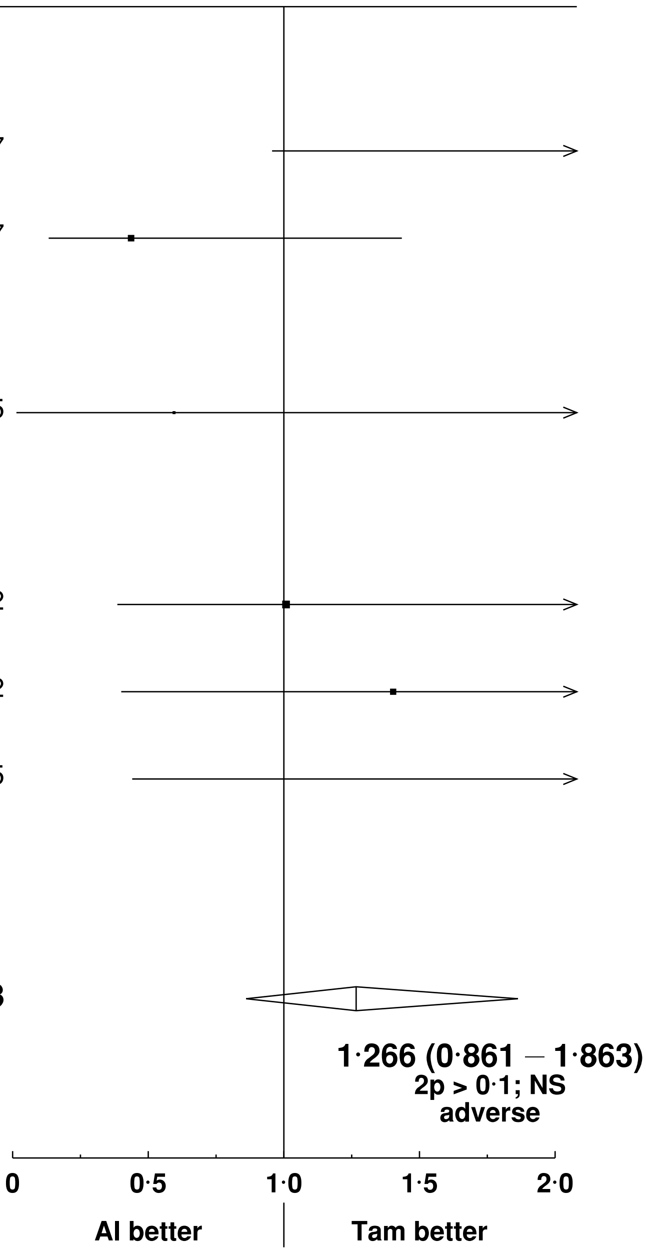

99% or 95% confidence intervals

Heterogeneity between 5 categories: χ^2_4 = 10·6; p = 0·03

Treatment effect 2p > 0·1; NS, adverse

P18: 5 year cumulative risk recurrence by (A) death without recurrence, (B) endometrial cancer incidence, (C) bone fracture incidence

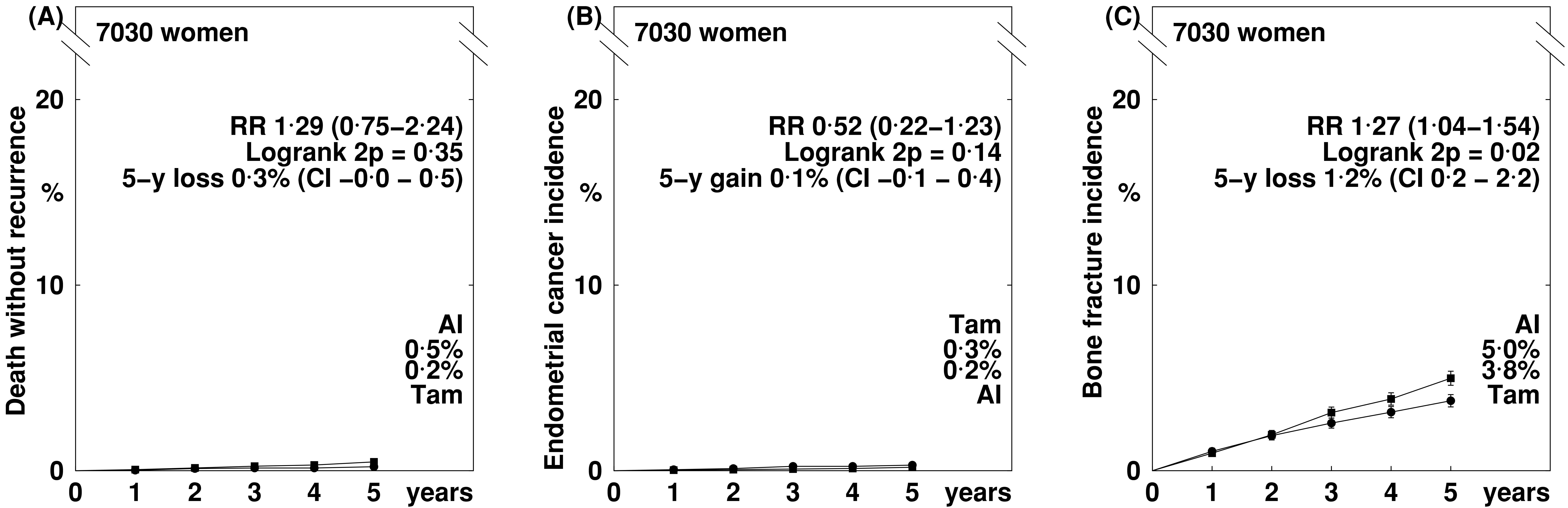

Death–without–recurrence rates (% / year) and logrank analyses

Allocation	Years 0 – 1	Years 2 – 4	Year 5+
AI	0·07 (5 / 6854)	0·11 (10 / 9421)	0·16 (15 / 9476)
Tam	0·06 (4 / 6805)	0·03 (3 / 9152)	0·19 (17 / 9098)
Rate ratio, from	1·29 CI 0·35 – 4·78	2·94 CI 0·99 – 8·73	0·90 CI 0·44 – 1·86
(O–E) / V	0·6 / 2·2	3·5 / 3·2	–0·7 / 7·4

Event rates (% / year) and logrank analyses

Allocation	Years 0 – 1	Years 2 – 4	Year 5+
AI	0·03 (2 / 6898)	0·04 (4 / 9669)	0·01 (1 / 9882)
Tam	0·06 (4 / 6868)	0·06 (6 / 9600)	0·05 (5 / 9827)
Rate ratio, from	0·51 CI 0·10 – 2·56	0·67 CI 0·19 – 2·32	0·32 CI 0·05 – 1·84
(O–E) / V	–1·0 / 1·5	–1·0 / 2·5	–1·4 / 1·2

Bone fracture incidence rates (% / year) and logrank analyses

Allocation	Years 0 – 1	Years 2 – 4	Year 5+
AI	0·98 (67 / 6853)	1·05 (99 / 9425)	0·65 (61 / 9356)
Tam	0·95 (65 / 6816)	0·65 (61 / 9416)	0·57 (54 / 9471)
Rate ratio, from	1·02 CI 0·73 – 1·44	1·62 CI 1·18 – 2·21	1·16 CI 0·80 – 1·68
(O–E) / V	0·8 / 32·4	18·9 / 39·3	4·2 / 28·4

Pages 19-20: Published adverse events in trials of AI verse tamoxifen in pre-menopausal women (% of women, unless otherwise stated)

Year code	Trial name	N	Bone fracture	Non-fatal cardiac events	Thrombosis or embolism	Stroke	Uterine	Compliance	Follow-up (median)
99B	ABCSG XII	1803	Ana vs Ana+Z vs Tam vs Tam+Z: *<br>• Fracture: 0.2 vs 0 vs 0.2 vs 0.2; p=0.91<br>• Serious Fracture: 0.9 vs 1.6 vs 1.3 vs 0.9; p=0.75	Ana vs Ana+Z vs Tam vs Tam+Z: *<br>• Tachycardia: 1.1 vs 2.2 vs 0.4 vs 2.0; p=0.07<br>• Serious tachycardia: 0.2 vs 0.2 vs 0.2 vs 0.0; p=1.00	Ana vs Ana+Z vs Tam vs Tam+Z: *<br>• Thrombosis: 0.0 vs 0.2 vs 0.0 vs 0.0; p=0.50<br>• Serious thrombosis: 0.0 vs 0.0 vs 0.7 vs 1.1; p=0.01	Not reported	Ana vs Ana+Z vs Tam vs Tam+Z: *<br>• Uterine polyp: 0.2 vs 0.2 vs 0.0 vs 1.1; p=0.07<br>• Serious uterine polyp: 1.5 vs 1.1 vs 8.9 vs 11.4; p<0.001	Number of patients who 'did not comply with treatment':<br>Ana vs Ana+Z vs Tam vs Tam+Z: 5 v 2 v 0 v 2<br>Patient compliance with endocrine therapy was confirmed during the 3-year treatment phase:<br>• Ana alone: in 4362 (89%) of 4884 visits<br>• Ana+Z: in 4769 (98%) of 4886 visits<br>• Tam alone: in 4492 (93%) of 4823 visits<br>• Tam + Z: in 4450 (93%) of 4778 visits	47.8 months
03E<br>03<	SOFT<br>TEXT	3066<br>2672	Ex+OS vs Tam vs Tam+OS: ¶<br>• any: 7.7 vs 5.3 vs 6.0<br>• grade 3 or 4: 1.6 vs 0.8 vs 1.0	Ex+OS vs Tam vs Tam+OS: ¶<br>cardiac ischemia or infarction:<br>• any: 0.7 vs 0.5 vs 0.4<br>• grade 3 or 4: 0.3 vs 0.4 vs 0.3<br>(One patients in the Tam group in SOFT had a grade 5 event)	Ex+OS vs Tam vs Tam+OS: ¶<br>• any event: 1.2 vs 2.2 vs 2.3<br>• grade 3 or 4: 0.9 vs 1.7 vs 2.0	Ex+OS vs Tam vs Tam+OS: ¶<br>CNS cerebrovascular ischemia:<br>• any event: 0.3 vs 0.6 vs 0.4<br>• grade 3 or 4: 0.2 vs 0.4 vs 0.3<br>CNS haemorrhage:<br>• any event: 0.8 vs 1.5 vs 1.1<br>• grade 3 or 4: <0.1 vs 0.0 vs 0.1	‡ Ex+OS vs Tam+OS<br>Gynaecological cancer: 0.3 (7/2346) vs 0.4 (9/2344), including endometrial cancer: 0.1 (2/2346) vs 0.2 (5/2344)	Exe+OS vs Tam vs Tam+OS: ¶<br>Discontinuation of endocrine therapy (with/without alternative therapy): 23.7 vs 22.5 vs 19.3	9 years
04]	HOBOE	483	No bone fracture.	Let vs Tam:<br>• Arrhythmia:<br>- any: 3.3 vs 3.1<br>- severe: 0 vs 0<br>• Cardiac ischemia:<br>- any 0.3 vs 0;<br>- severe 0.3 vs 0.<br>• Cardiovascular – other:<br>- any: 2.2 vs 3.1<br>- severe: 0 vs 0	Let vs Tam:<br>• any: 0 vs 0.3<br>• severe: 0 vs 0	Let vs Tam:<br>Central nervous system cerebrovascular ischemia:<br>• any: 0 vs 0.6<br>• severe: 0 vs 0.6	Not reported	Let vs Tam:<br>• Treatment stopped because of toxicity: 6.7 vs 5.6<br>• Treatment stopped because of refusal: 0.6 vs 1.7<br>• Treatment completed per protocol: 59.3 vs 49.2	64 months

Abbreviations: Exe=exemestane. N=number of women randomised (unless otherwise specified). Let=Letrozole. OS= ovarian suppression. Tam=tamoxifen. Z=zoledronic acid.

Data in the table is from most recent publication unless '‡' indicated then from a previous publication

* P values are for a four-group comparison according to Fisher's exact test. Serious adverse events: any adverse events that were lethal or life-threatening, resulted in permanent damage, required inpatient hospitalization or extension of inpatient treatment, or placed the patient at risk and necessitated medical or surgical intervention.

† Data based on the 4643 patients in the safety population in SOFT and TEXT who received a protocol-assigned treatment.

¶ Data based on safety populations in SOFT and TEXT who initiated a protocol-assigned treatment, including 1005 patients who were randomly assigned to receive tamoxifen in SOFT and 4643 patients who were randomly assigned to receive tamoxifen plus ovarian suppression (2326 patients) or exemestane plus ovarian suppression (2317 patients) in SOFT or TEXT

Reference

Year code	Trial name	Reference
99B	ABCSG XII	Gnant M, Mlineritsch B, Schippinger W, Luschin-Ebengreuth G, Pöstlberger S, Menzel C, Jakesz R, Seifert M, Hubalek M, Bjelic-Radisic V, Samonigg H. Endocrine therapy plus zoledronic acid in premenopausal breast cancer. *N Engl J Med*. 2009;360(7):679-691.<br>Gnant M, Mlineritsch B, Stoeger H, et al. Adjuvant endocrine therapy plus zoledronic acid in premenopausal women with early-stage breast cancer: 62-month follow-up from the ABCSG-12 randomised trial. *Lancet Oncol* 2011;12(7):631-41.
03E<br>03<	SOFT<br>TEXT	Pagani O, Regan MM, Walley BA, Fleming GF, Colleoni M, Láng I, Gomez HL, Tondini C, Burstein HJ, Perez EA, Ciruelos E. Adjuvant exemestane with ovarian suppression in premenopausal breast cancer. *N Engl J Med*. 2014;371(2):107-118.<br>Francis PA, et al. Tailoring adjuvant endocrine therapy for premenopausal breast cancer. *N Engl J Med*. 2018;379(2):122-137.
04]	HOBOE	Nuzzo F, et al. Bone effect of adjuvant tamoxifen, letrozole or letrozole plus zoledronic acid in early-stage breast cancer: the randomized phase 3 HOBOE study. *Ann. Oncol*. 2012;23(8):2027-2033.<br>Perrone F, et al. Adjuvant zoledronic acid and letrozole plus ovarian function suppression in premenopausal breast cancer: HOBOE phase 3 randomised trial. *Eur J Cancer*. 2019;118:178-186.

Pages 21-29: Statistical Analysis Plan

DRAFT Statistical Analysis Plan for a Meta-Analysis of trials of Aromatase inhibitors versus tamoxifen in premenopausal ER+ women with ovarian suppression
November 11th 2021, Version 1.2

Objective
To collate individual patient data to characterise the benefits and risks of trials comparing aromatase inhibitors (AI) with tamoxifen in premenopausal ER+ women with ovarian suppression.

Analyses
Primary analyses will be by Intention-to-Treat (ITT), including all randomised patients irrespective of treatment compliance.

Sensitivity analyses will also be undertaken to investigate the potential impact of non-compliance on treatment efficacy.

The primary analyses will be of the following comparisons (trials included in each section are listed in appendix 1):

- Trials of AI versus tamoxifen in premenopausal ER+ women with operable breast cancer who have undergone ovarian ablation or ovarian suppression

Primary outcomes
The main endpoint definitions and analyses methods are those used in previous EBCTCG reports, with amendments reflecting the potential impact of endocrine therapy.

1. **Time to recurrence:** includes distant recurrence, local recurrence and new second primary breast cancer (ipsilateral or contralateral), and the definitions of these will be as in each trial.
2. **Breast cancer mortality:** Information about mortality rates without recurrence will be subtracted from information about overall mortality rates (as in previous EBCTCG reports). The same statistical methods will be used to construct Kaplan-Meier graphs that estimate breast cancer mortality (i.e. the pattern of mortality that would have been seen if it had been possible to avoid all deaths before or after recurrence from causes other than breast cancer).
3. **All-cause mortality**

Exploratory endpoints

1. **Time to first distant recurrence:** includes distant recurrence and ignores any prior loco-regional or contralateral recurrences. Information about the site(s) of first distant recurrence will be collected to allow additional exploratory analyses to be performed. Patients can have more than one site recorded as their first distant recurrence. Categorisation of sites will include, but is not limited to: liver, lung, bone, node, soft tissue, brain.
2. **Time to loco-regional recurrence as first event**: includes ipsilateral breast, chest wall and locoregional lymph nodes (axilla and SCF).
3. **Time to new contralateral breast cancer**
4. **Death without recurrence:** i.e. without the secretariat having any record of recurrence. Because the quality of the recurrence data in the AI trials is likely to be reasonably good, deaths from a wholly unknown cause without record of recurrence will be treated as deaths from an unknown cause that was not breast cancer.
5. **New primary endometrial cancer rates:** Endocrine therapy with tamoxifen is associated with an increased risk of endometrial cancers though aromatase inhibitors may reduce the risk of endometrial cancer.
6. **Fracture rates:** Information on fractures, including date of first fracture and site where available will be collected.

7. **Safety –** Data on other non-fatal adverse events in individual trials (e.g. ischemic heart disease, stroke, pulmonary embolus, DVT) will be sought and analysed.
8. **Incidence and site of second cancers** – Quality assurance checks will be undertaken to compare the incidence of second cancers (overall and by site) with that of distant recurrence by age group to characterise whether these are true second cancers or miscoded recurrences. Where there is confidence that second cancers are correctly diagnosed, the incidence of second cancers before any recurrence of breast cancer will be provided by treatment group for each cohort. In addition, second cancers will be categorised by site.

Subgroup analyses:

Exploratory subgroup analyses will be undertaken but, given the well-known hazards of subgroup analysis, will be interpreted appropriately cautiously. Investigation of potential interactions between tumour or patient characteristics and treatment efficacy will be undertaken with breast cancer recurrence as primary outcome. However, if almost all of the benefits of AI over tamoxifen are seen in the period when the treatments differed, as they were in the EBCTCG meta-analysis of AI vs tamoxifen in postmenopausal women (Lancet 2015), then subgroup analyses of recurrence just in this period will also be undertaken to enhance the statistical power to investigate any variability in treatment efficacy by patient or tumour characteristics.

Subgroup analyses of Recurrence Forest plots for subgroup analyses by:

- Site of recurrence (distant metastasis, local recurrence or contralateral breast cancer)
- Age (<45, 45-54, 55-69, ≥70, unknown),
- PR status (PR-, PR+, PR unknown)
- HER2:CEP17 ratio (HER2-, HER2+, HER2 unknown)
- Nodal status (N0, N1-3, N4+, N unknown)
- Tumour stage (T1, T2, T3/T4, T stage unknown);
- Histological grade (1, 2, 3, unknown),
- Proliferation index (%Ki-67: 0-9, 10-19, 20+, unknown)
- Tumour histology (ductal, lobular, other, unknown)
- Presence/absence of chemotherapy
- Period of follow-up years 0-1, 2-4, 5-9, and 10+ after randomisation

Tests of heterogeneity and of trend

First calculate the log-rank statistic (o-e) and its variance v in each separate stratum, and add these up to get the overall logrank (O-E) and it variance V (i.e. the sum of the separate variances). Delete any uninformative strata (i.e. those for which v is zero), and number the remaining strata from 1 to n. A χ^2 test (on n-1 degrees of freedom) for heterogeneity between the treatment effects in different strata can be obtained by subtracting $(O-E)^2/V$ from the sum of the separate values, one per stratum, of $(o-e)^2/v$.

Alternatively, a χ^2 test for trend (i.e. for whether the treatment effect changes progressively from one stratum to the next) will be calculated as follows: if the stratum numbered s has logrank statistics (o-e) and v then define m, the mean stratum number, to be the sum, one term per stratum, of sv/V and define T to be the sum, one term per stratum, of (s-m)(o-e). The variance of T, var(T), is then the sum, one term per stratum, of $(s-m)^2v$, and the χ^2 test (on 1 degree of freedom) for trend is $T^2/var(T)$. If there are only two strata then the tests for trend and heterogeneity are identical.

Appendix 1. Trial list

Trial (Yr) Code	Trial name	Comparison	Size	Data status
**AI vs tamoxifen in premenopausal women**				
2618/23 (99B)	ABCSG XII, Austria	Goserelin; (Anastrozole vs tamoxifen) ± Zoledronic Acid 3yr	1803	Received
7629/36 (03<)	TEXT/IBCSG 25-02, Switzerland	Triptorelin; (Exemestane vs tamoxifen) 5yr	2639	Received
7627 (03E)	SOFT/IBCSG 24-02, Switzerland	Triptorelin; (Exemestane vs tamoxifen) 5yr	3066	Received
21204 (04])	IT Naples HOBOE, Italy	Triptorelin; (Letrozole vs tamoxifen) 5yr	1065	Received

Appendix 2.

EBCTCG seventh cycle variables and data format

Either using the codes we suggest below or using your own codes, please extract from your dataset the variables that correspond most closely to the items listed below and send them to us. Please provide one record for each person ever randomised (including any person who was randomised and then was later categorised as ineligible, withdrawn, unevaluable, lost or "protocol deviant"—but, please tell us in question 9 which patients your group's preferred analyses would exclude, and why).

For trials where a dataset has previously been sent to the EBCTCG it is probably easiest and most reliable to update by re-sending all variables. If, however, this would cause difficulties then you can send only the additional variables; let us know if you want a file of the data you previously sent and we will provide it.

If any variable is not available or not applicable, please omit it and send only the remaining variables. If you have any of the requested variables in your records in a form that would require substantial additional work to supply (e.g. computerisation, or manual coding), feel free to omit them for now, but in your cover document please tell us of their existence. Please send the following:

- Your data in a separate Excel spreadsheet for each separate trial, if possible.
- A cover document giving all your coding conventions (including your format for dates).
- Send data to: bc.overview@ndph.ox.ac.uk with your research group's name (and/or the EBCTCG number for your research group) and your group's name for the trial in the subject line.

If you have any questions about this data request, please contact the EBCTCG secretariat on bc.overview@ndph.ox.ac.uk (Telephone: +44-1865-743852). All data supplied to the secretariat will be held securely and treated confidentially, in accordance with the EBCTCG Data Policy (available at: https://www.ctsu.ox.ac.uk/research/ebctcg).

For additional notes on supplying data to the overview, please see the EBCTCG Collaborators' Space web pages, or email the EBCTCG secretariat

CORE VARIABLES—BASELINE (Q1–27)

A) RANDOMISATION AND PATIENT CHARACTERISTICS (Q1–9)

1. **Your patient identifier** *(preferably specifying uniquely which trial as well as which patient)*
2. **Date of randomisation** *(specify your format for dates [in your covering document])*
3. **Allocated treatment** *(specify your codes)*
4. **Age** at randomisation *(years)* **NB Here & everywhere else, leaving an item Blank means Not Known**
5. **Height** at randomisation *(m)*
6. **Weight** at randomisation *(kg)*
7. **Menopausal status** at randomisation *(1=pre-, 2=peri-, 3=postmenopausal with intact ovaries & uterus, 4=ovarian ablation, 5=hysterectomy, 6=both [ie, 4 and 5]), 7=artificial, 8=male patient)*
8. **Did chemotherapy cause apparently permanent cessation of menses?** *(1=no/not applicable, 2=yes)*
9. **Would your group's preferred analyses exclude this patient?** NB A few trial patients may be randomised in error, otherwise ineligible, lost with no follow-up, unevaluable or withdraw consent. *(1=no known reason for exclusion, 2=yes [specify main reason(s) for preferring exclusion, if known])*

B) SURGICAL DETAILS (Q10–11; OR, DEFINE AND USE YOUR OWN CODES)

10. **Breast surgery** *(1=none, 2=only lumpectomy or wide local excision, 3=quadrantectomy or sector resection, 4=partial mastectomy, 5=simple or total mastectomy, 6=radical mastectomy, 7=modified radical mastectomy)*
11. **Axillary surgery** *(1=none, 2=sentinel node biopsy only, 3=axillary sampling, 4=surgical clearance of less than levels I & II, 5=full clearance of axillary levels I & II, 6=clearance of more than levels I & II, 7=axillary clearance, but levels cleared unspecified)*

C) PATHOLOGICAL NODAL STATUS (Q12–13; OR, USE YOUR OWN CODES [EG, TNM])

Note: In patients receiving neo-adjuvant treatment (or in trials where neo-adjuvant or axillary treatment differs between groups) give nodal status prior to neo-adjuvant (or axillary) therapy in section K

12. **Sentinel node biopsy** *(1=not done; 2=done and negative for cancer; 3=no greater involvement than isolated tumour cells [≤0.2 mm and/or < 200 cells]; 4=no greater involvement than micrometastases [>0.2mm or >200cells but ≤2mm]; 5=macroscopic nodal deposits [>2 mm]; 6=positive but size unknown)*
13. **Axillary status** *(specify codes, or: 1=pN- histologically; 2=N- other/unknown method; 3=1-3 positive nodes ; 4=4-9 [or 4+] positive; 5=10+ positive; 6=N+ histologically, unknown number; 7=N+ other/unknown method)*

D) TUMOUR CHARACTERISTICS (Q14–18; OR, USE YOUR OWN CODES [EG, TNM])

14. **Method first detected** *(1=mammographic screening, 2=incidental, 3=symptomatic, 4=other)*
15. **Laterality** *(1=left, 2=right, 3=bilateral)*
16. **Pathological grade prior to any neo-adjuvant therapy** *(1=well differentiated, 2=moderately,3=poorly)*
17. **Histological type** (if not locally determined please state)*(1=invasive, not otherwise specified, 2=ductal, 3=lobular, 4=other invasive, 5=mixed, 6=carcinoma in situ (CIS) only)*
18. **Tumour diameter:** largest diameter of excised primary *(mm)*

E) RECEPTOR STATUS (Q19–27; OR, USE YOUR OWN CODES)

Note: In trials with some neo-adjuvant treatment give receptor status prior to any neo-adjuvant therapy

19. **Summary of Estrogen Receptor (ER) status of primary tumour** *(1=ER-poor, 2=ER+, 3=ER++ [define in cover document, unless ER-poor is <10 fMol/mg and ER++ is ER definitely ≥100 fMol/mg])*
20. **Quantitative ER measurement** *(measured in central/reference lab if possible, otherwise best available)*
21. **Units for ER** *(1=fMol/mg, 2=% +ve by IHC, 3=Allred score [category score], 4=H-score, 9=other [specify]*)

22. **Summary of Progesterone Receptor (PR) status of primary tumour** *(1=PR-poor, 2=PR+, 3=PR++ [define in cover document, unless PR-poor is <10 fMol/mg and PR++ is PR definitely ≥100 fMol/mg])*
23. **Quantitative PR measurement** *(done in central/reference lab if possible, otherwise best available)*
24. **Units for PR** *(coded as Q21)*

25. **Summary of HER2 status of primary** *(1=negative/normal, 2=positive/over-expressing)*
26. **Quantitative HER2 measurement** *(done in central/reference lab if possible, otherwise best available)*
27. **Units for HER2** *(1=IHC [% staining], 2=IHC score [0, 1+, 2+, 3+], 3=FISH [# copies], 4=FISH [HER2:CEP17 ratio], 5=CISH [# copies], 6=CISH [HER2:CEP17], 9=other [please specify])*

F) NON-COMPLIANCE BEFORE ANY RECURRENCE (Q28–29; OR, USE YOUR OWN CODES)

28. **Any substantial deviation from trial treatment allocation (before any breast cancer recurrence)?** *(1=no, 2=never started, 3=discontinued, 4=switched to opposite trial group, 5=other [specify])*
29. **Date of first such deviation from allocated treatment** *(ignore deviations after recurrence)*

G) CANCER RECURRENCE AND SECOND CANCERS (Q30–40; OR, USE YOUR OWN CODES)

30. **Any recurrence of invasive breast cancer (ie, locoregional, contralateral or distant)?** NB Includes any occurrence of new ipsilateral or contralateral breast cancer *(1=no, 2=yes)*
31. **<u>If no:</u> Date patient last known to be free of such recurrence; <u>If yes:</u> Date of first such recurrence**

32. **Site of first distant recurrence (ie, possibly distant; not just locoregional/contralateral)** *(1=no distant recurrence, 2=recurrence-unknown if distant, 3=distant recurrence-unknown site(s), 4=only in distant soft tissue, 5=only in distant nodes, 6=only in bone, 7=only visceral, 8=only in CNS, 9=multiple sites including bone but not CNS/brain, 10=multiple sites not including bone or CNS/brain, 11=multiple sites including CNS/brain but not bone, 12=multiple sites including CNS/brain and bone)*
33. **Date of first distant recurrence** NB Locoregional recurrence can precede first distant recurrence

34. **Site of first locoregional recurrence** *(1=no locoregional recurrence recorded, 2=multiple or unspecified locoregional sites 3=only in breast [new or recurrent invasive cancer] or chest wall, 4=only in axilla, 5=only in other locoregional nodes [eg, infraclavicular fossa], 6= multiple locoregional sites, 7=only in internal mammary nodes, 8=only in supraclavicular nodes, 9=tumour bed, 10=breast but known not tumour bed, 11=CIS only (if index CIS or if subsequent invasive cancer not collected))*
35. **Date of first locoregional recurrence**

36. **Contralateral breast cancer?** *(1=no, 2=yes: new invasive cancer thought to have arisen during follow-up in the contralateral breast, 3=CIS only (if index CIS))*
37. **Date of first contralateral breast cancer**
    NB If patient had more than one second malignancy during follow-up, <u>repeat</u> variables 37-39 for each.
38. **Site of any second malignancy *[except breast cancer (including contralateral)]* during follow-up** *(Describe ALL sites. Use and specify your own codes; if you use ICD codes specify revision, eg ICD-9 or ICD-10)*
39. **Date of this second malignancy**
40. **MIGHT this have been a breast cancer metastasis?** *(1=no, 2=possibly/not yet certain [eg, possible lung, liver, bone or brain metastasis: please do not report definite breast metastases as second cancers])*

H) SURVIVAL (Q41–43)

41. **Is patient known to have died?** *(1=no, 2=yes)*
42. **<u>If NO:</u> Date patient last known to be alive; <u>If yes:</u> Date of death**
43. **<u>If YES:</u> Cause of death** *(use and specify your own codes; if you use ICD codes specify which version, eg ICD-9 or ICD-10)*

ADDITIONAL VARIABLES (Q44–59)

I) *ADDITIONAL TUMOUR MARKER DATA (Q44–52; OR, USE YOUR OWN CODES)*

Note: If tests of gene expression or special tests of IHC quantitation were done on the excised primary then please send a separate file in your own format with the fully detailed set of results on each individual.

44. **Summary of gene-expression status of primary tumour** *(1=low risk, 2=intermediate risk, 3=high risk): NB Please also provide the fully detailed gene expression results for each patient as a separate dataset.*

45. **Quantitative gene-expression prognostic score** *(best available single numerical measure)*

46. **Prognostic score used to quantify gene expression profile** *(use own code, or: 1=OncotypeDx prognostic score, 2=Mammaprint prognostic score, 3=EndoPredict, 4=Prosigna,9=other [please specify])*

47. **Summary of Topo-isomerase II alpha (TOPO2A) status of primary tumour** *(1= normal [ie, no gene over-expression or deletion], 2=positive/over-expressing, 3=deleted)*

48. **Quantitative TOPO2A measurement** *(done in central/reference laboratory if possible)*

49. **Units for TOPO2A** *(1=IHC [% staining], 2=IHC score [0, 1+, 2+, 3+], 3=FISH [number of copies], 4=FISH [TOPO:CEP17 ratio], 5=CISH [# copies], 6=CISH [TOPO:CEP17], 9=other [please specify])*

50. **Summary of Proliferation Index of primary tumour** *(1=low, 2=intermediate, 3=high)*

51. **Quantitative Proliferation Measure** *(best available numerical measure, in central/ ref lab if possible)*

52. **Factor measured for Proliferation Index** *(1=S-phase fraction [%], 2=thymidine labelling index [%], 3=Ki-67 by IHC [% staining], 9=other [please specify]*)

J) *NON-FATAL ADVERSE EVENTS (Q53–54; OMIT IF NOT SOUGHT)*

Note: Some treatments may cause or prevent bone fractures, cardiovascular events, lymphoedema, or lung fibrosis. Please describe all such events (eg, hip fracture, spinal fracture, myocardial infarction, stroke, pulmonary embolus, episode of cardiac failure) if, but only if, such events were sought and recorded systematically for all arms of the trial.

If more than one such event was recorded, repeat variables 52-53 for each.

53. **Nature and severity of event** *(use your own codes; if you use ICD codes, specify which version, eg ICD-9 or ICD-10, and if you use CTC Adverse Event codes, please specify version number, eg CTCAE-3 or CTCAE-4)*

54. **Date of event**

K) *TRIALS WITH SOME NEO-ADJUVANT SYSTEMIC THERAPY OR WHERE AXILLARY TREATMENT DIFFERS BETWEEN GROUPS (Q55–59; OR, USE OWN CODES)*

55. **Apparent axillary nodal status (clinical, radiological or other) before neo-adjuvant** *(1=N-, 2=N+)*

56. **Apparent tumour diameter (clinical or radiological) before neo-adjuvant**: largest diameter *(mm)*

57. **Operability before any neo-adjuvant therapy** *(define your own codes, or: 1=Breast-conserving surgery feasible, 2=Mastectomy but not BCS feasible, 3=inoperable, 4=uncertain operability)*

58. **Breast tumour response after completion of neo-adjuvant** *(define your own codes, or: 1=clinically complete response [cCR] & negative pathology (for invasive disease and DCIS), 2=cCR with DCIS, 3=cCR with invasive cancer remaining pathologically, 4=cCR with no pathological information, 5=partial response, 6=stable disease, 7=progression [define 5–7])*

59. **Axillary response after neo-adjuvant** *(coded as Q58)*

ADDITIONAL VARIABLES FOR SPECIFIC META-ANALYSES (Q60–87)

For some meta-analyses we may need additional information on surgery, radiotherapy, adjuvant treatments received, or additional tumour markers but this section does not need to be completed for most meta-analyses so omit unless specifically requested.

L) *TRIALS OF EXTENDED ENDOCRINE THERAPY (Q60–63)*

Note: This information is needed only for trials of longer versus shorter endocrine therapy

60. **Endocrine therapy given prior to randomisation** *(1=Tamoxifen, 2=Aromatase Inhibitor, 3=Tamoxifen then Aromatase Inhibitor, 4=Aromatase Inhibitor then Tamoxifen, 5=Other (specify))*
61. **Date initial endocrine therapy started** *(Approximate date, or date of surgery if unknown)*
62. **Date of first switch from tamoxifen to AI, or vice versa** *(if applicable)*
63. **Date initial endocrine therapy completed** *(Approximate date)*

M) *DETAILS OF ADDITIONAL TREATMENTS FOR TRIALS OF LOCAL, BIOLOGICAL, OR ENDOCRINE THERAPY (Q64–68)*

64. **Neoadjuvant chemotherapy received** *(1=no, 2= non-anthracycline, non-taxane, 3=anthracycline, non-taxane, 4=taxane+anthracycline, 5=other taxane-containing, 9=Yes – type unknown)*
65. **Adjuvant chemotherapy received** *(as for Q64)*
66. **Endocrine therapy received** *(1=no, 2=tamoxifen, 3=aromatase inhibitor, 4=sequential tamoxifen and aromatase inhibitor, 5=ovarian ablation/suppression alone, 6=ovarian ablation+tamoxifen, 7=ovarian ablation + aromatase inhibitor, 9=Yes – type unknown))*
67. **HER2 directed therapy received** *(1=no, 2=Yes)*
68. **Radiotherapy received (irrespective of site)** *(1=No, 2=Yes)*

N) *ADDITIONAL THERAPY DETAILS FOR TRIALS OF LOCAL THERAPY (Q69–79)*

69. **Date of first breast surgery** *(dd/mm/yyyy)*
70. **Site of tumour in breast quadrant** *(1=lateral, 2=medial, 3=central, 4=medial or central, 5=not specified)*
71. **Lymphovascular invasion** *(1=no, 2=yes)*
72. **Date of first axillary surgery** *(dd/mm/yyyy)*
73. **Total number of sentinel lymph nodes excised and examined pathologically** *(-1=none, 1=one, 2=two, etc)*
74. **Number of sentinel lymph nodes excised and were:**
    a. **isolated tumour cells [≤0.2 mm and or ≤200 cells]** *(-1=none, 1=one, 2=two, etc)*
    b. **micrometastasis (>0.2mm or >200cells but ≤2mm)** *(-1=none, 1=one, 2=two, etc)*
    c. **macroscopic nodal deposits [>2 mm]** *(-1=none, 1=one, 2=two, etc)*
    d. **positive (unknown whether ITC, micro or macrometastasis)** *(-1=none, 1=one, 2=two, etc)*
    e. **negative** *(-1=none, 1=one, 2=two, etc)*

75. **Radiotherapy to whole breast/chest wall** *(1=No, 2=Yes)*
76. **Radiotherapy to partial breast** *(1=No, 2=Yes)*
77. **Radiotherapy to supraclavicular fossa** *(1=No, 2=Yes)*
78. **Radiotherapy to axilla** *(1=No, 2=Yes)*
79. **Radiotherapy to internal mammary chain** *(1=No, 2=Yes)*

O) *ADDITIONAL DETAILS FOR TRIALS OF DCIS (Q80–84)*

80. **Closest relevant excision margin** *(please give in mm where available; 99=no excision performed)*
81. **Diagnosis at entry** *(1=DCIS only, 2=DCIS+LCIS, 3 = DCIS (+/- LCIS) with microinvasion, 4=unknown)*
82. **Comedo** *(1=Present, marked or severe; 2=Present, moderate; 3=Present, slight; 4=Present, NOS; 5=Absent; 6=Unknown)*
83. **Architecture** *(1=Cribriform; 2=Micropapillary; 3=Papillary; 4=Solid; 5=Other (please specify), 6=Unknown)*
84. **Focality** *(1=Unifocal;2=Multifocal/multicentric;3=Unknown)*

P) ADDITIONAL QUESTIONS FOR TRIALS OF DURATION OF BIOLOGICAL THERAPY *(Q85–88)*

Note: This information is needed only for trials of longer versus shorter biological therapy (e.g. trastuzumab duration)

85. Biological therapy given prior to randomisation *(1=None, 2=Yes)*
86. Date initial biological therapy started *(Approximate date, or date of surgery if unknown)*
87. Date initial biological therapy completed *(Approximate date)*

Q) FURTHER TUMOUR MARKERS *(Q88–91)*

88. Summary of Tumour Infiltrating Lymphocytes (TILS) status *(1=low, 2=intermediate, 3=high)*
89. Quantitative TILS Measure *(% staining positive, in central/ ref lab if possible)*
90. Central histological type *(coded as for Q17)*
91. E-Cadherin data *(1=negative, 2=positive,3=intermediate/unclear)*
